# Current Status and Challenges in Identifying Disease Resistance Genes in *Brassica napus*

**DOI:** 10.3389/fpls.2017.01788

**Published:** 2017-11-06

**Authors:** Ting Xiang Neik, Martin J. Barbetti, Jacqueline Batley

**Affiliations:** ^1^School of Biological Sciences, University of Western Australia, Perth, WA, Australia; ^2^School of Agriculture and Environment and Institute of Agriculture, University of Western Australia, Perth, WA, Australia

**Keywords:** *Brassica napus*, *R* gene, genomics, host-pathogen interaction, qualitative resistance, pathotype

## Abstract

*Brassica napus* is an economically important crop across different continents including temperate and subtropical regions in Europe, Canada, South Asia, China and Australia. Its widespread cultivation also brings setbacks as it plays host to fungal, oomycete and chytrid pathogens that can lead to serious yield loss. For sustainable crop production, identification of resistance (*R*) genes in *B. napus* has become of critical importance. In this review, we discuss four key pathogens affecting *Brassica* crops: Clubroot (*Plasmodiophora brassicae)*, Blackleg (*Leptosphaeria maculans* and *L. biglobosa)*, Sclerotinia Stem Rot (*Sclerotinia sclerotiorum*), and Downy Mildew (*Hyaloperonospora parasitica)*. We first review current studies covering prevalence of these pathogens on *Brassica* crops and highlight the *R* genes and QTL that have been identified from *Brassica* species against these pathogens. Insights into the relationships between the pathogen and its *Brassica* host, the unique host resistance mechanisms and how these affect resistance outcomes is also presented. We discuss challenges in identification and deployment of *R* genes in *B. napus* in relation to highly specific genetic interactions between host subpopulations and pathogen pathotypes and emphasize the need for common or shared techniques and research materials or tighter collaboration between researchers to reconcile the inconsistencies in the research outcomes. Using current genomics tools, we provide examples of how characterization and cloning of *R* genes in *B. napus* can be carried out more effectively. Lastly, we put forward strategies to breed resistant cultivars through introgressions supported by genomic approaches and suggest prospects that can be implemented in the future for a better, pathogen-resistant *B. napus*.

## Introduction

*Brassica napus* is cultivated worldwide for its edible oil (*B. napus* var. *oleifera* or *napus*; oilseed rape), as a vegetable for human consumption (*B. napus* var. *pabularia*; Siberian or rape kale) or animal fodder (*B. napus* var. *napobrassica*; rutabagas, swedes). Its economic importance across some of the major production countries/regions is highlighted in Table 1.

**Table 1:** Economic importance of *B. napus*.

**Year**	**Country/Region**	**Economic value ($m or $b pa) and/or area of cultivation/production (kha)**	**Harvested production (1,000 tons)**	**References**
2014–2016	Australia	AU$1.7 b/2,160 kha	3,435	Australian Bureau of Statistics, 2016; Australian Oilseeds Federation, 2016
2017	Canada	$15.4 b (between years 2007–2010)/9,059 kha	2,100	Rempel et al., 2014; Statistics Canada, 2017
2016	France	€1,935 m/2,234 kha	6,200	Eurostat, 2016
2014	China (rapeseed)	US$3.6 billion^*^/7,534 kha	14,931	National Bureau of Statistics of China, 2015

* *Economic benefits of importing Canadian canola*.

With intensified *B. napus* cultivation in many countries, to meet the demands for oil and vegetable production, the incidence and severity of disease caused by various pathogens has also increased (Sanogo et al., 2015; Van de Wouw et al., 2016a). The major diseases and key pathogens that cause serious damage on *B. napus* worldwide include the chytrid Clubroot pathogen (*Plasmodiophora brassicae*); fungal pathogens such as Sclerotinia Stem Rot (*Sclerotinia sclerotiorum*), Blackleg (*Leptosphaeria maculans, L. biglobosa*), White Rust (*Albugo candida*), Light Leaf Spot (*Pyrenopeziza brassicae*), Alternaria Blight (*Alternaria brassicae, A. brassicicola*, other *Alternaria* spp.) and White Leaf Spot (*Pseudocercosporella capsellae*); the oomycete Downy Mildew pathogen (*Hyaloperonospora parasitica*), and the bacterial Blackrot pathogen (*Pseudomonas syringae*).

Fungicidal or cultural control measures offer inconsistent and expensive disease management options, especially for low input and low return cropping systems. Effective host resistance is the most cost-effective and reliable means of disease control and resistance(s) have been identified for all major diseases; including race/pathotype-dependent and -independent resistances. Many of these pathogens do not restrict themselves to *B. napus* but infect, to varying degrees, different *Brassica* crop species (Ren et al., 2016). As such, the great genetic diversity within and between the different *Brassica* crop species and other non-*Brassica* species within the Brassicaceae provides a rich resource for resistance (*R*) genes against the different major pathogens.

*B. napus* (AACC, *n* = 19) is an amphidiploid, formed by interspecific hybridization of the two diploid species *B. rapa* (AA, *n* = 10) and *B. oleracea* (CC, *n* = 9). During this hybridization, several rearrangement events occurred that resulted in extensive reordering among the diverged genomes (Truco et al., 1996). This is supported by the recently published genome sequences of *B. rapa* (Wang et al., 2011; Cai C. et al., 2017), *B. napus* (Chalhoub et al., 2014), *B. oleracea* (Parkin et al., 2014; Liu S. et al., 2014), *B. juncea* and *B. nigra* (Yang et al., 2016) and the pan-genome of *B. oleracea* (Golicz et al., 2016). The A and C genomes in *B. napus* are highly homologous, due to their common ancestry (Liu S. et al., 2014). Evidence of the A and C genomes sharing high genome similarity is also provided from common ancestral blocks between *B. rapa* and *B. oleracea* (Parkin et al., 2005, 2014; Schranz et al., 2006).

Plants recognize pathogens either through the pathogen's pathogen-associated molecular patterns (PAMPs) with the plant's pattern recognition receptors (PRRs) or the pathogen's virulence molecules, termed effectors, interacting with the plant *R* genes (Jones and Dangl, 2006). Although different definitions of *R* genes have been put forward (Kushalappa et al., 2016), we associate *R* genes with the conventionally described version that is a single/major gene that results in qualitative resistance and is highly specific (Chisholm et al., 2006; Bent and Mackey, 2007). In contrast, quantitative resistance involves more than one gene, which often lie within a major QTL region that displays large phenotypic variance (Remington and Purugganan, 2003). The specificity between the host *R* gene and the pathogen effector happens only for pathogens that have adapted to the plant. This plant immunity is termed effector-triggered immunity (ETI), signified by localized cell death that constitutes a hypersensitive response (HR)—an “all-or-none” effect, which is often the exception rather than the rule (Dodds and Rathjen, 2010). Mostly, plants respond to pathogens by mounting resistance called PAMP-triggered immunity (PTI), which is a non-host resistance. Plants also defend themselves by triggering defense related hormone signaling pathways such as jasmonates (JA), salicylates (SA), ethylene (ET), abscisic acid (ABA), and brassinosteroids (BR) (Kazan and Lyons, 2014). This type of resistance is called systemic acquired resistance (SAR) and often incurs broad-spectrum and long lasting immunity (Durrant and Dong, 2004; Netea et al., 2011). Evidence shows that *R* gene mediated plant immunity and SAR involving defense response hormone signaling are tightly regulated (Larkan et al., 2013; Ma and Borhan, 2015) revealing the complexity of the plant immunity (Thomma et al., 2011; Stotz et al., 2014). In *Brassica* species, both major *R* genes and quantitative QTL have been identified for all key host pathogens. However, the number of these genes that have been characterized and cloned is very limited. This causes challenges for breeding for resistance and an increased reliance on chemical control. Current advances in genomics in *B. napus* have provided us with a good platform to search for candidate *R* genes using advanced molecular tools (Edwards et al., 2013; Zhang Y.-M. et al., 2016; Bayer et al., 2017).

Here, we review past and current studies on identification of *R* genes across a wide range of *Brassica* species, focusing on four major fungal pathogens and the diseases they cause, viz. Clubroot (*Plasmodiophora brassicae*), Blackleg (*L. maculans* and *L. biglobosa)*, Sclerotinia Stem Rot (*Sclerotinia sclerotiorum*), and Downy Mildew (*Hyaloperonospora parasitica)*. We ascertain sources of *R* genes based on genes mapped in *Brassica* hosts for each of these pathogens and discuss possibilities of these *R* genes for breeding resistant *B. napus* using genomics tools. We highlight challenges in identification and deployment of *R* genes, and discuss options and strategies for the most effective utilization of *R* genes to improve resistance to major fungal, oomycete or chytrid pathogens of *B. napus*.

## Sources of *R* genes for key pathogens

### Clubroot

**Figure 1:**
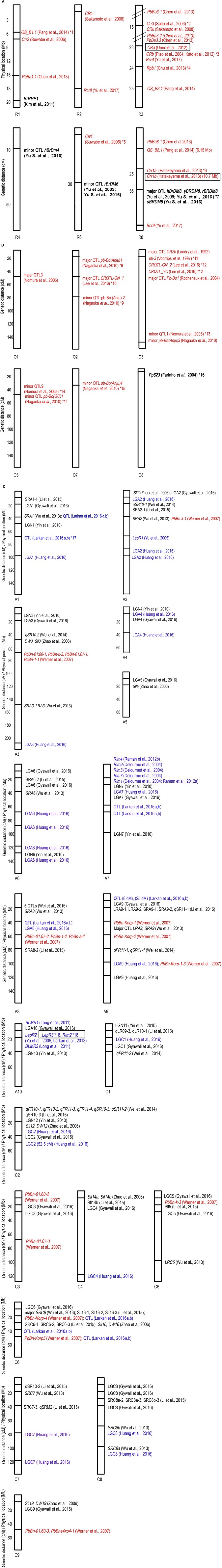
*R* genes/QTL mapped on *B. rapa* **(A)**, *B. oleracea* **(B)**, and *B. napus* **(C)**. Each vertical rectangular is a chromosome, with the chromosome name followed by chromosome number at the bottom. Black font denotes *R* genes/QTL mapped for *S. sclerotiorum*, blue font *L. maculans*, red font *P. brassicae* and bold black font *H. parasitica*. Boxed *R* gene is cloned. Where genetic distance applies, the chromosome position is stated in parentheses at the end of the locus. ^*^1, Independent minor loci, homologous to *A. thaliana* chromosome 3; ^*^2, *Crr3* is independent of *Crr1* and *Crr2;* ^*^3, Syntenic with *A. thaliana* chromosome 4. *Crr1* and *Crr2* also share the same syntenic region; ^*^4, Close to *CRa* and *CRb*, also known as *Rcr1* (Chu et al., 2014), predicted as TIR-NBS-LRR (Yu F. et al., 2016); ^*^5, Independent of *Crr1, Crr2*, and *Crr3*; ^*^6, Two genes, *Crr1a* and *Crr1b* were identified at originally single *Crr1* locus; ^*^7, All at the same locus and are identical to major QTL *BraDM* (Yu et al., 2009); ^*^8, Candidate gene, *Bra016457*, is serine/threonine (S/T) kinase. The major QTL in ^*^7 and ^*^8 lie between 17.6 and 17.8 Mb on chromosome R8; ^*^9, Syntenic with *CRc* on chromosome R2; ^*^10, Strong collinearity with chromosome A2 in *B. rapa* where *CRc* gene is located. Also overlap with *pb-Bo(Anju)1*; ^*^11, Identified by Voorrips et al. (1997) but mapped on chromosome O3 by Nagaoka et al. (2010); ^*^12, *CRQTL-GN_2* (15.5–24.3 cM) and *CRQTL_YC* (8.5–38.1 cM) have similar location. Syntenic regions for these two QTL are on chromosome R3 (*Crr3, CRk, CRa*, and *CRb*), R8 (*Crr1*); ^*^13, Same linkage group with QTL-LG1 (Moriguchi et al., 1999); ^*^14, May be the same locus; ^*^15, Closely linked to *CRb*, homologous to chromosome R3; ^*^16, Adult resistance; ^*^17, Field resistance for *L. maculans*. Candidate gene is cysteine rich RLK; ^*^18, *LepR3* and *Rlm2* are allelic variants.

*Plasmodiophora brassicae*, causal agent for Clubroot, has a wide host range including non-cruciferous plants (Ren et al., 2016). Clubroot has caused severe cruciferous crop yield loss of up to 50% in Canada (Strelkov and Tewari, 2005) and 20–30% in China (Chai et al., 2014). The pathogen physiology, progress in efforts to combat this disease, and an update on its epidemiology in Canada, Europe and China, have recently been the subject of various reviews (Chai et al., 2014; Diederichsen et al., 2014; Dixon, 2014; Strelkov and Hwang, 2014; Ríčarová et al., 2016). Furthermore, sources of *P. brassicae R* genes and genetic mapping studies have been reviewed by Piao et al. (2004); Rahman et al. (2014). To date, at least 19 *R* genes/QTL have been mapped for Clubroot resistance in *B. napus*, mostly from the study by Werner et al. (2007). However, only two Clubroot resistance genes have been cloned, *Crr1a* and *CRa*, both in *B. rapa* (Table 2 and Figure 1).

**Table 2:** Key pathogens of Brassica crops and R genes/QTL that have been genetically mapped or cloned in relation to *Plasmodiophora brassicae, Sclerotinia sclerotiorum, Hyaloperonospora parasitica*, and *Leptosphaeria maculans*.

**Nature of pathogen and disease caused**	**Resistance gene/QTL mapped**	**Resistance gene/allele cloned**
*Plasmodiophora brassicae*, an obligate biotroph chytrid (protista) causing Clubroot disease	- *B. napus* (Canola lines “1CA0591.323” and “1CA0591.263” (Resistant, R) derived from cultivar “Mendel” × “A7-26NR” (Susceptible, S), using isolate SACAN-ss1, Williams pathotype 3): “Mendel” carries *CRa* and *CRb^*Kato*^* on chromosome A3 (Fredua-Agyeman and Rahman, 2016). *CRa* and *CRb^*Kato*^* may be same allele as also shown in Zhang T. et al. (2014) - *B. napus* (Inbred line “12-3” (R) × inbred line “12-1” (S) using pathotype 3): New CR gene, act singly or combined with *CRa* (Zhang H. et al., 2016) - *B. napus* (DH “263/11” (R) × Cultivar “Express” (S), 7 different isolates from Sweden, Germany, and France): 19 QTL mapped. Broad-spectrum resistance mapped on chromosome A2, A3, A8, A9, C3, C5, C6, and C9. QTL on A3 corresponds to the region for *CRk* and *Crr3*, some QTL have additive effects (Werner et al., 2007) - *B. napus* (Cultivar “Mendel”(R) × Breeding line (S), using isolate 1 that is highly virulent on *B. oleracea*): One single dominant locus from *B. rapa* (“ECD-04”), two recessive loci from *B. oleracea* (“ECD-15”) (Diederichsen et al., 2006) - *B. napus* (DH progeny from Darmor-*bzh* (R) × Yudal (S), using single spore isolate pathotypes 4 and 7 according to (Somé et al., 1996)): Monogenic or polygenic depending on isolate, major resistance dominant gene *Pb-Bn1* toward isolate Pb137-522 on LG DY4, also show weaker partial resistance effect in association with other QTL. Additive and epistatic QTL were identified (Manzanares-Dauleux et al., 2000) - *B. napus* (Natural lines *B. oleracea* ”ECD-15” (R) × *B. rapa*” ECD-04” (R)), from which cultivar “Mendel” is produced, using virulent field isolates): Two dominant, unlinked genes (Diederichsen and Sacristan, 1996) - *B. rapa* (Fine map *Rcr2* in Chinese cabbage cv. “Jazz” (R) × DH ACDC (S) using pathotype 3): *Rcr2* on 22 and 26 Mb of chromosome A3 (Huang et al., 2017) - *B. rapa* (Breedin*g* line “T19” originate from cultivar “Pluto” (R) × DH line ”ACDC” (S), using Williams pathotype 2,3,5,6,8, 5x): GBS identified *Rcr4* on chromosome A3 and two novel QTL, *Rcr8* on A2 and *Rcr9* on A8 (Yu et al., 2017) - *B. rapa* (Inbred lines Chinese cabbage “CCR13685” (R) × Pak choi “GHQ11021” (S), using isolate with unknown race/pathotype): single dominant gene (Chen et al., 2016) - *B. rapa* (Fine map *Rpb1* from Chu et al., 2013): Single dominant allele *Rcr1* (or also known as *Rpb1*) on LG A3, close position to both *CRa* and *CRb*, against Williams pathotype 3 (Chu et al., 2014). *Rcr1* is predicted to be TIR-NBS-LRR (Yu et al., 2017) *- B. rapa* (Pak choi “702-5” (S) × DH line “CR Shinki” (R), using Williams race 4): Mapped physical location of *CRb* on chromosome A3 on *B. rapa* with interval length of 83.5 kb with 15 candidate genes including NBS-LRR genes. *CRb* is not an allele of *CRa, but closely linked genes. CRa and CRb^Kato^ may be same allele as shown in* Fredua-Agyeman and Rahman (2016) and Zhang T. et al. (2014) - *B. rapa* (Inbred lines turnip “Siloga” (R) × Chinese cabbage “BJN3” (S), using isolate Williams race 4): One major locus *QS_B3.1* on chromosome A3 corresponding to *CRa* and *CRb*, and two minor loci *QS_B1.1* on chromosome A1 (homologous to *Arabidopsis* chromosome 3 independent of any other CR loci) and *QS_B8.1* on chromosome A8 (share same locus with *Crr1b* and *Crr1*). Additive effects and epistatic interactions were found in *B. rapa* (Pang et al., 2014) - *B. rapa* (Five Chinese cabbage cultivars (R) × *B. rapa* oilseed accession (S) using Canadian field isolate): linkage group N3 corresponding to chromosome A3 (Gao et al., 2014) - *B. rapa* (Fine map *CRb* in DH line “CR Shinki” (R) × Chinese cabbage “702-5” (S) using pathotype 4): *CRb* gene was tightly linked to two other *CR* genes, *CRa* and *CRb^*Kato*^* (Zhang T. et al., 2014) - *B. rapa* (DH “G004” (R) x DH “A9709” (S), using field isolates Ano-01, Wakayama-01, and Nos. 5, 7, 9, and 14 based on Hatakeyama et al. (2004): *Two* genes, *Crr1a* with major effect and *Crr1b* with minor effect at *Crr1* locus (Hatakeyama et al., 2013) - *B. rapa* (Inbred turnip line “ECD04” (R) × Inbred Chinese cabbage line “C59-1” (S) × using four different isolates Pb2, Pb4, Pb7, and Pb10): Partial resistance, *PbBa1.1 on A1, PbBa3.1, PbBa3.2 and PbBa3.3* on A3 (*PbBa3.1* and *PbBa3.3* on different region), *PbBa8.1* on A8 (Chen et al., 2013) - *B. rapa* (Cultivar Pak choi “FN” (R) x DH “ACDC” (S), using isolate Williams pathotype 2, 3, 5, 6, 8): Single dominant gene *Rpb1* located on LG A3, close to *CRa* (Chu et al., 2013) - *B. rapa* (Cultivars “Akiriso” and “CR Shinki,” using isolate No. 14 or pathotype group 3 according to Hatakeyama et al., 2004): Single dominant gene linked to *CRb*, or is *CRb* found in “Akiriso” (Kato et al., 2012). *CRb* and *CRa* are one and the same clubroot-resistance gene (Hatakeyama et al., 2017) - *B. rapa* (Fine map *Crr1* in *Arabidopsis*): *Crr1* is likely consist of two genetic loci. The gene order is conserved except for one inversion in which insertion is found (Suwabe et al., 2012) - *B*. rapa (Mapping of *CRa* from (Matsumoto et al., 1998)): RFLP marker, HC352b closely linked to *CRa* (Hayashida et al., 2008) - *B. rapa* (Two F2 populations, one F2 progeny from DH “K10” (R) × DH “Q5” (S), the other DH “C9” (R) × DH “6R” (S), using isolate M85 Williams race 2 and K04 with unknown pathotype/race): Two loci: *CRk* and *CRc*, broad spectrum resistance. *CRk* is very near or identical to *CRb* on chromosome A3. *CRc* is mapped on chromosome A2 or R2 (Sakamoto et al., 2008). - *B. rapa* (Doubled haploid, DH “G004” (R) × DH “A9709” (S), using isolate Wakayama-01 which has not been classified but is close to Williams race 4): Two loci, *Crr1* and *Crr2*, polygenic control, both loci needed for resistance. *Crr1* is closely linked to the major QTL toward Williams race 2 in Kuginuki et al. (1997) (Suwabe et al., 2003) - *B. rapa* (Map position of *Crr1* and *Crr2* on *A. thaliana* and *B. rapa*): *Crr1* is on linkage group LG7 (chromosome A8 or R8) and *Crr2* on LG6 (chromosome A1 or R1) of *B. rapa*, both aligned to same region of chromosome 4 in *Arabidopsis* (Suwabe et al., 2006) - *B. rapa* (Map position of *Crr3* on *B. rapa* and *Arabidopsis* from Hirai et al., 2004): *Crr3* is on R3, same LG as *CRb*, syntenic region on *Arabidopsis* chromosome 3 (Saito et al., 2006) - *B. rapa* (DH Chinese cabbage “CR Shinki” (R) × “94SK” (S), using isolate Williams race 4): Single dominant gene, *CRb* (Piao et al., 2004) that is closely linked to *CRa* on chromosome A3 or R3 (Diederichsen et al., 2009). *CRb, Crr1*, and *Crr2* fall in the same syntenic region of *Arabidopsis* chromosome 4 (Saito et al., 2006) - *B. rapa* (DH “G004” (R) × DH “A9709” (S), using isolate Wakayama-01 and Ano-01): *Crr4* loci on LG 2 (chromosome A6 or R6), independent of *Crr1, Crr2*, and *Crr3* (Suwabe et al., 2006) - *B. rapa* (Inbred line “N-WMR-3” containing cultivar “Milan White” (R) × DH “A9709” (S), using isolate Ano-01 Williams race 3): *Crr3*, major dominant gene, independent of *Crr1* and *Crr2* (Hirai et al., 2004) - *B. rapa* (DH “T136-8” (R) × DH “Q5” (S), both Chinese cabbage, using isolate Williams race 2): Single dominant major gene, *CRa* (chromosome A3 or R3) (Matsumoto et al., 1998) - *B. rapa* (European fodder turnip “Siloga S2” (R) × DH Chinese cabbage “Homei” (S), using isolate Williams race 2): Single dominant phenotype plus heterogeneous phenotype (Kuginuki et al., 1997) - *B. oleracea* (Korean inbred cabbage lines “C1220” (R) and “C1176” (S): Three QTL, *CRQTL-GN_1* (chromosome C2) and *CRQTL-GN_2* (chromosome C3), using GN isolate Williams race 9) and *CRQTL-YC* (chromosome C3), using YC isolate Williams isolate race 2): *pB-Bo (Anju)1* and *pB-Bo (Anju)3* overlap with *CRQTL-GN_1* and *CRQTL-GN_2* (or *CRQTL-YC*) respectively. *CRQTL-YC* is major QTL for race 2 and also minor QTL for race 9 isolates. Both closely linked QTL *CRQTL-GN_2* and *CRQTL-YC* have syntenic regions on *B. rapa* chromosome A3 (*Crr3, CRk, CRa*, and *CRb)* and A8 (*Crr1)* (Lee et al., 2016) - *B. oleracea* (DH cabbage “Anju” (R) × DH broccoli “GC” (S) using isolate Williams race 4): *pb-Bo(Anju)1* and *pb-Bo(Anju)2* on chromosome O2 (syntenic R2 where *CRc* is detected), *pb-Bo(Anju)3* on chromosome O3 (corresponds to R3 distal end), *pb-Bo(Anju)4* on chromosome O7 (closely linked to *CRb*, homologous to R3), *pb-Bo(GC)1* on chromosome O5. Polygenic control for “Anju” resistance with *pb-Bo (Anju)1* being major QTL while the rest is moderate and minor QTL. Reported that *CR2b* and *pb-3* are also on chromosome O3 (Nagaoka et al., 2010) - *B. oleracea* (Inbred kale line “K269” (R) × Inbred cabbage line “Y2A” (S), using isolate Williams race 4): Three QTL namely QTL 1, 3, and 9 with complementary effect (Nomura et al., 2005). QTL 9 and *PbBo(GC)1* may be same locus (Nagaoka et al., 2010) - *B. oleracea* (Landrace kale line “C10” (R) × DH broccoli “HDEM” (S), using isolate pathotype according to (Somé et al., 1996) Ms6 (P1), K92-16 (P4), Pb137-522 (P7), eH (P1), K92 (P2)): Nine QTL, major QTL *Pb-Bo1* (isolate specific) against three out of five isolates (Pb137-522, K92-16, and K92), minor QTL (non-specific) *Pb-Bo3, Pb-Bo4, Pb-Bo8, Pb-Bo9a, Pb-Bo9b for isolates* Pb137-522, K92-16, K92, eH, and Ms6 respectively (Rocherieux et al., 2004) - *B. oleracea* (Inbred kale “K269”(R) × Inbred cabbage “Y2A” (S), using field that is infected with isolate Williams race 1 and 3): One major QTL on LG 3 (Moriguchi et al., 1999) - *B. oleracea* (German landrace “Bindsachsener” (R) × DH broccoli “Greenia” (S), using isolate ECD 16/3/30): *pb-3* and *pb-4* additive effect on LG 3 and 1 respectively (Voorrips et al., 1997) - *B. oleracea* (Kale line “C10” (R) × Cauliflower line “48.4.7” (S), using isolate Williams race 7): Polygenic control (Grandclément and Thomas, 1996) - *B. oleracea* (Kale breeding lines, using isolate Williams race 4): Young and adult stage resistance was assessed. Dominant alleles, additive effects and incomplete dominance were found (Laurens and Thomas, 1993) - *B. oleracea* (Inbred broccoli “CR7” (R) × Cultivar cauliflower “Early White” (S), using isolate Williams race 7): Mixed dominant QTL and polygenic QTL (Figdore et al., 1993) - *B. oleracea* (European Clubroot Differential or ECD host set including cabbage, broccoli, kale, using Dutch isolate): Display recessive character (Voorrips and Visser, 1993) - *B. oleracea* (Cabbage line “No. 86-16-5” (R) × rapid cycling line “CrGC No. 85”, using isolate Williams race 2): Two dominant QTL, *CR2a* (derived from *B. napus*), *CR2b* on LG 6 and 1, respectively (Landry et al., 1992)	- *Crr1a* (TIR-NB-LRR) from *B. rapa* derived from European fodder turnip cultivar “Siloga” resistant to isolate Ano-01 Williams race 3 (Hatakeyama et al., 2013) - *CRa* (DH lines “T136-8” and “Q5”, Chinese cabbage cultivar “Ryutoku” and “CR Shinki”, fodder turnip “Debra” and “Gelria R”, using M85 clubroot isolate) (TIR-NB-LRR) from *B. rapa* (Ueno et al., 2012)
*Sclerotinia sclerotiorum*, a necrotrophic fungal pathogen causing Sclerotinia Stem Rot disease	- *B. napus* (152 accessions from Canada, China and Europe, as well as South Korea and Japan, using isolate #321 collected from oilseed rape fields in Alberta, Canada): 21 loci conferring resistance to *S. sclerotiorum* and 13 loci conferring susceptibility mapped to 12 of the 19 *B. napus* chromosomes (Gyawali et al., 2016) - *B. napus* (347 Chinese accessions comprising spring, winter and semi-winter lines, using same isolate in (Mei et al., 2013): 17 SR QTL on chromosome A8 and chromosome C6 including five on A8, and 12 on C6. The C6 QTL corresponds to C6 in (Zhao et al., 2006; Wu et al., 2013; Li et al., 2015)). Candidate genes were predicted based on GWAS and transcriptome sequencing (Wei L. et al., 2016) - *B. napus* (Physical map construction based on *B. napus* genome): 35 QTL mapped including eight LR and 27 SR. LR QTL distributed across chromosome A9 (corresponding to A9 region in Wu et al. (2013), C1 and C9, SR QTL mapped to 12 chromosomes (A1, 2, 6, 8, 9, C2, 4, 5, 6, 7, 8, 9) where C9 corresponds to that in (Mei et al., 2013). Some SR and LR QTL share same genomic region (Li et al., 2015) - *B. napus* (DH population from cultivar “Express” (female) × Chinese line “SWU7” (male), using same isolate in Mei et al. (2013)): Six field resistance (FR) QTL and five SR under controlled environment identified. Four FR and two SR mapped to chromosome C2 and one SR on chromosome A2, where C2 is homoeologous to A2 (corresponding to chromosome N2 and chromosome N12 in Zhao et al. (2006) and both chromosomes syntenic with progenitor genomes (Wei et al., 2014) - *B. napus* (Pure line “J7005” (R) × Cultivar “Huashang 5” (S), using Chinese isolate): 13 QTL identified. Three LR on LG A3, A9, and C5, SR on LG A1, 2, 3, 6, 8, 9, C6, and C7, C8. Two Major QTL are *LRA9* and *SRC6* (LG A9 and LG C6 respectively); both quantitative traits with additive gene effects. *BnaC.IGMT5.a* is a candidate gene for QTL *SRC6* (Wu et al., 2013) - *B. napus* (DH population from line”DH821” (R) × line “DHBa0604” (S), using Chinese isolate): 21 QTL, on chromosome N3 (or A3), chromosome N4 (or A4), chromosome N11 (or C1), chromosome N17 (or C7), chromosome N12 (or C2) (Yin et al., 2010) - *B. napus* (Two DH populations: Chinese winter line “Hua dbl2” (R) × European spring line “P1804” and Cultivar “Major” (R) × Cultivar “Stellar” (S), using isolate 105HT derived from the US soybeans): Nine QTL on seven LGs including chromosome N2 (or A2) with its homeologous non-reciprocal transposition on chromosome N12 (or C2), chromosome N16 (or C6), chromosome N5 (or A5), chromosome N14 (or C4), chromosome N3 (or A3), chromosome N19 (or C9) (Zhao et al., 2006) - *B. napus* (Breeding line “Ning RS-1” (R) × male sterility restorer line “H5200,” using isolate HY-12): Six QTL including three LR QTL and three mature stage resistance with one major QTL *qLRS1* on LG 17 and one major QTL, *qSRM1* on LG 15 with additive effect (Zhao and Meng, 2003) - *B. oleracea* (Wild relative of *B. oleracea* that is *B. incana* (R) × Cultivated *B. oleracea* var. *alboglabra* (S), using same isolate in Mei et al., 2013): Multiple epistatic interactions (polygenic genes) for LR and SR, control of these genes is different in both resistances (Disi et al., 2014) - *B. oleracea* (Wild relative of *B. oleracea* that is *B. incana* (R) × Cultivated *B. oleracea* var. *alboglabra* (S), using Chinese field isolate): 12 QTL for leaf resistance (LR) and 6 QTL for stem resistance (SR). Two major QTL on chromosome C9 for LR and SR with corresponding region on chromosome A9 (Mei et al., 2013)	None
*Hyaloperonospora parasitica* (syn. *Peronospora parasitica*), an obligate biotroph oomycete causing Downy Mildew disease	- *B. napus* (Resistant accessions “RES-26,” “RES-02,” and Susceptible cultivar “Callypso,” using isolates R1 and P003 provided by University of Nottingham): Single, partially dominant gene in RES-26 and two independent partially dominant genes in RES-02. These resistant genes could be closely linked, allelic or identical. Another single, incomplete dominant gene is found in RES-02 (Nashaat et al., 1997) - *B. napus* (Cultivar “Victor,” “Jet Neuf,” and “Cresor,” using isolate R3 collected from winter oilseed rape crop in Leicestershire in 1982): Single dominant gene (Lucas et al., 1988) - *B. rapa* (Mapping population derived from (Yu et al., 2009): Four major QTL including *sBrDM8* (seedling resistance, identical to *BraDM), yBrDM8* (young plant), *rBrDM8* (rosette), and *hBrDM8* on chromosome A8 and two minor QTL *rBrDM6* on chromosome A6 and *hBrDM4* on chromosome A4. Candidate gene for *sBrDM8* is serine/threonine kinase (STK) family (Yu S. et al., 2016). - *B. rapa* (Inbred lines “RS1” (R) × “SS1” (S), using natural infection in Korea): Single dominant gene *BrRHP1* on LG A1 (Kim et al., 2011) - *B. rapa* (DH population from line “T12-19” × line “91-112” (S), using Chinese isolate): Major QTL *BraDM* for seedling resistance on LG A8 and minor QTL on A6 (Yu et al., 2009). SSR markers for *BraDM* were developed by Yu et al. (2011) - *B. oleracea* (BAC libraries BoT01 and BoCig): Single dominant locus *Pp523* on chromosome C8 and C5 (Carlier et al., 2011) - *B. oleracea* (Mapping population of Portuguese genotypes “Couve Algarvia,” “Penca de Chaves,” and “Couve de Corte” and “DH-GK97362” (S), using Portuguese isolate P501): Two dominant genes at cotyledon stage and a single dominant gene at adult stage, inheritance is independent of stage (Monteiro et al., 2005) - *B. oleracea* (52 entries including landraces, wild accessions and hybrid between wild and cultivars, using mixture of field isolates from *B. napus* in Sweden): Recessive resistance gene at cotyledon stage (Carlsson et al., 2004) - *B. oleracea* (S_4_ line derived from accession OL87125 (R) × rapid-cycling *B. oleracea* DH line “GK97362” (S), using unknown isolate source): *Pp523* locus on chromosome C8 confers adult resistance (Farinhó et al., 2004) - *B. oleracea* (DH broccoli “USVL089” (R) × DH “USVL047” (S), using US field isolate): Single dominant gene at cotyledon stage (Farnham et al., 2002) - *B. oleracea* (DH broccoli “USVL012” (R) × DH “USVL047” (S), using the US field isolate): Two unlinked dominant genes at true leaf stage (seedling resistance) (Wang et al., 2001) - *B. oleracea* (DH broccoli “USVL” series (R) and hybrid “Green Valiant” (S), using isolate SC1 that is race 2 isolate as described by (Thomas and Jourdain, 1990) and CA1 from broccoli field in California with unknown race specificity): Polygenic (Wang et al., 2000) - *B. oleracea* (Cauliflower F_1_ hybrids 9304F1, 9305F1, 9306F1, 9311F1, and two open-pollinated cultivars “Perfection” and “Aberdeen,” using Denmark isolate FYN93.cau and others from Europe, UK and the US): Single dominant gene at cotyledon stage, associate with adult stage (Jensen et al., 1999a) - *B. oleracea* (20 DH broccoli from “Corvet,” “Shogun,” “Skiff,” “Atsumori,” and “OSU” series, using three isolates, FYN82.cau, Lincs82.cau, and Moz82.cab obtained from cauliflower or broccoli host from Europe, UK, US and Mozambique): Partial resistance at cotyledon stage (Jensen et al., 1999b) - *B. oleracea* (Cauliflower cc (R) × HR 5-4 (S), cc (R) × 244 (S), 3-5-1-1 (R) × 244 (S), cc (R) × 3-5-1-1 (R) and 244 (S) × 267-6-9 (S) in India, using natural infection method): Single dominant gene *PPA3* with recessive epistasis (Mahajan et al., 1995) - *B. oleracea* (10 broccoli breeding lines, using isolates from the US): Single dominant gene and modifying genes at seven or more leaf stage (Dickson and Petzoldt, 1993) - *B. oleracea* (Broccoli and cauliflower accessions from the US Plant Introduction collection, using US isolate): Single major gene against race 2 (Thomas and Jourdain, 1990) - *B. juncea* (RESBJ accessions from Canada, Germany, and China, using 12 isolates from the UK and India derived from *B. juncea, B. rapa* and *B. napus*): Accessions RESBJ-200 and RESBJ-190 conditioned by single dominant genes, different in each accession but recognizing same isolate (Nashaat et al., 2004) - *B. juncea* (31 spring type *B. juncea* accessions, susceptible control is winter type *B. napus* cv. *Ariana*, using four isolates from the UK and India derived from *B. napus* and *B. juncea*): mostly resistant with homozygous and heterozygous resistance (Nashaat and Awasthi, 1995)	None
*Leptosphaeria maculans*	- *B. napus* (GWAS panel of 179 accessions from DH population SAgS described in Raman R. et al. (2016), evaluated for resistance against 12 single spore isolates): Major *R* gene for adult plant resistance *Rlm12* on chromosome A1 (Raman H. et al., 2016) - *B. napus* (DH populations from cultivar “AG-Castle” and “AV-Sapphire” (R) × “Topas” (S), field experiment in Australia): Three QTL for adult plant resistance on chromosome A1, A8, A9, and C6 where candidate genes include cysteine-rich receptor-like kinases on A1 (Larkan et al., 2016a) - *B. napus* (DH lines from BnaDYDH mapping population derived from “Darmor-*bzh*”(R) × “Yudal” (S) and developed in France, field experiments in the UK and France): 17 QTL for adult plant resistance across 13 LGs (Huang et al., 2016) - *B. napus* (Worldwide accessions from Germplasm Resources Information Network, using PG-4 isolate): one major QTL on chromosome A1 (Rahman M. et al., 2016) - *B. napus* (“DH12075” derived from cultivar “Cresor” that has *R* gene *LmR1* × Westar, (S) using natural ascospores released from infected stubble): *LepR4* recessive on A genome (Yu et al., 2013) - *B. napus* (186 DH population SASDH, derived from *Rlm4* cultivar “Skipton” and “Ag-Spectrum,” using 11 single spore isolates from the national blackleg isolate collection in Australia): Single major gene *Rlm4* mapped on chromosome A7 (Raman et al., 2012b). Characterization of *Rlm4* candidate genes in the same population (Tollenaere et al., 2012) - *B. napus* (DH “Maxol” and “Columbus”): Mapped *Rlm1* on chromosome A7 (Raman et al., 2012a) - *B. napus* (SASDH population derived from “Skipton”/“Ag-Spectrum,” using Australian isolates): *Rlm4* major qualitative locus mapped on chromosome A7 (Raman et al., 2012b) - *B. napus* (Mapping populations of cultivar “Surpass 400” (R) × “Westar” (S), using isolate 87-41): *BLMR1* and *BLMR2*, single major gene on chromosome N10 (Long et al., 2011) - *B. napus* (Two different mapping populations,”DH12075” from cultivar “Cresor” (R) × re-synthesized line “PSA12” (S) and “Shiralee” (R) × “PSA12” (S), using unknown source of isolate): *ClmR1* same genetic interval as *LmR1* on chromosome A7 (Mayerhofer et al., 2005) - *B. napus* (Mapping population of cultivars carrying published *Rlm* gene, using isolate PHW1245 (IBCN74) and IBCN56): *Rlm9* single gene control (Delourme et al., 2004) - *B. napus* (Cultivar “Surpass 400,” using 31 isolates from Canada, Australia, Europe, Mexico and USA comprising PG2-4): *LepR3* single dominant allele, same linkage group as *LepR2* on the A genome (Li and Cowling, 2003; Yu et al., 2008) - *B. napus* (DH population, “DHP95” and “DHP96” with resistance introgressed from *B. rapa* subsp. *sylvestris*, using 30 isolates from Canada, Australia, Europe, and Mexico): *LepR1* (complete, inhibit growth) and *LepR2* (incomplete, reduced growth) on A genome chromosome A2 and A10 respectively (Yu et al., 2005) - *B. napus* (Cultivars based on published differential set, using isolates from France, Australia, New Zealand, England and Portugal): *Rlm3, Rlm7* single gene control (Balesdent et al., 2002) - *B. napus* (DH and F_2:3_ populations from “Darmor” (R) × “Samourai” (S), field experiment in France): 16 genomic regions for field resistance (Pilet et al., 1998, 2001) - *B. napus* (Cultivar “Doublol,” “Vivol,” “Columbus,” and “Capitol,” “Jet Neuf,” using isolate PG2-4): *Rlm4* linked to *Rlm1* (Balesdent et al., 2001) - *B. napus* (DH from cultivar “Maluka,” “Cresor,” and “RB87-62” × “Westar” (S), using isolate PG2): *cRLMm, cRLMrb* cited in single resistance gene at cotyledon stage and, *aRLMc* and *aRLMrb* adult stage linked to *cRLMm* and *cRLMrb* (Rimmer et al., 1999) - *B. napus* (Cultivar “Westar,” “Quinta,” and “Glacier,” using isolate PG2, PG3, and PG4): *Rlm1* single dominant gene (Ansan- Melayah et al., 1995; Ansan-Melayah et al., 1998) - *B. napus* (Cultivar “Westar,” “Quinta,” and “Glacier,” using isolate PG2-4): *Rlm2* single dominant gene (Ansan-Melayah et al., 1998) - *B. napus* (DH population from cultivar “Shiralee” and “Maluka” (R) × advanced breeding lines (S), using five single spore virulent isolates collected from provinces in Canada): *LmR1* single major locus, could be linked/identical (Mayerhofer et al., 1997) - *B. napus* (DH population from cultivar “Major” (R) × “Stellar” (S), using isolate PHW1245): *LEM1* single major locus (Ferreira et al., 1995) - *B. napus* (DH from cultivar “Cresor” (R) × “Westar” (S), using canola residues infected with virulent *L. maculans* and pycnidiospores of isolate Leroy): *LmFr1* single major gene (Dion et al., 1995) - *B. rapa* (Accession “02-159-4-1” (R) × DH “Z1” (S), and with “Darmor” and “Eurol,” using 31 isolates from the IBCN and IMASCORE collections): *Rlm11* single gene introgressed into *B. napus* (Balesdent et al., 2013) - *B. rapa* (Line “156-2-1”): *Rlm8* single control (Balesdent et al., 2002) - *B. juncea* (Cultivar “Aurea” and “Picra”): *Rlm5* and *Rlm6* epistatic interaction (Balesdent et al., 2002) - *B. juncea* (F2 population from F1 progeny of Cultivar “AC Vulcan” × Inbred line “UM3132,” using PG2 isolate): Two independent genes, one dominant and one recessive (Christianson et al., 2006)	-*LepR3* (Larkan et al., 2013) -Allelic variant *Rlm2* (Larkan et al., 2015)

Sources of *P. brassicae R* genes in *B. rapa* have been particularly promising as there are many race-specific, single, dominant *R* genes present, as compared with *B. oleracea* which has a more continuous resistance profile involving various combinations of both major and minor genes (Diederichsen et al., 2009; Rahman et al., 2011). Due to the different physiological resistance mechanisms toward *P. brassicae* in *B. rapa* and *B. oleracea*, complete resistance is often reported in the former but not in the latter (Tanaka et al., 2006; Donald et al., 2008; Hwang et al., 2012). Recent screening efforts in China have identified *B. rapa* to have good resistance against Canadian pathotype 3 (Zhang et al., 2015). Hence, it is not uncommon that breeding efforts make use of *B. rapa*, in particular using the European fodder turnip as the recurrent parent, to introduce resistance into *B. rapa, B. napus*, and *B. oleracea* (Diederichsen et al., 2009). The introgressed regions can be characterized using SNP genotyping and next generation sequencing in order to identify and characterize these resistance genes and determine if they are the same gene or distinct. They could then be deployed in a more targeted manner.

In Canada, most oilseed *B. napus* shows little, or at best only moderate, resistance to Williams pathotypes 3 and 5, while *B. napus* rutabaga lines have better resistance (Jakir Hasan et al., 2012). However, in Australia, the majority of *B. napus* was found to be resistant (Donald et al., 2006), reflecting the influence of the local abiotic environments on the pathogenicity of the fungus, or the origin of host and/or pathogen influencing the resistance outcome (Donald et al., 2006; Donald and Porter, 2014). Further QTL or Genome-Wide Association Study (GWAS) studies should be performed in *B. napus* in order to identify these resistance genes.

Traditionally, the AB genome amphidiploid *B. juncea* does not show high resistance. It often shows low, moderate (Peng et al., 2014) or intermediate (Sharma et al., 2012) resistance. However, more recently, one accession of *B. juncea* was found to be highly resistant to the local pathotype 4 under field conditions in China (Ren et al., 2016). Another good source of resistance from the B genome is diploid *B. nigra*, which has been reported to display high resistance against all local pathotypes in Canada (Jakir Hasan et al., 2012; Chu et al., 2013; Peng et al., 2014). There is therefore the potential for all A, B and C genomes of the *Brassica* species to harbor *R* genes against *P. brassicae*.

### Sclerotinia stem rot

Similar to the Clubroot pathogen, the Sclerotinia Stem Rot pathogen *Sclerotinia sclerotiorum* also has a wide host range, including members outside the crucifer family (Zhao et al., 2004). In the *Brassica* species, this pathogen can cause up to 80% yield losses in China (Mei et al., 2011) and has previously been reported to cause a loss of AU$ 23 million to the canola industry in Western Australia (Dafwa, 2015). The resistance studies on Sclerotinia Stem Rot have mainly focused on the inheritance type at different developmental stages; namely seedling leaf and adult stem stages of the host plant. Thus far, no *R* genes have been cloned for this pathogen (Wu et al., 2016b). However, candidate *R* genes against *S. sclerotiorum* have been identified and physically mapped on the *B. napus* genome (Li et al., 2015). These are NBS-LRR genes with gene copies found on chromosomes A6 and C7 (Li et al., 2015). Candidate *R* genes against *S. sclerotiorum*, belonging to the CC-NBS-LRR class, were also identified on chromosome A9 in *B. napus* (Mei et al., 2013). Yet, the majority of the QTL for *S. sclerotiorum* have been identified in the C genome (C9 and C6) (Li et al., 2015), indicating that *B. oleracea* is a good source of *R* genes for *S. sclerotiorum*. This was recently confirmed for many *B. oleracea* var. *capitata* genotypes from China (You et al., 2016).

There can be strong correlation between leaf and stem resistance across some specific *Brassica* genotypes (Mei et al., 2011; Wu et al., 2013). For example, a few Chinese *B. oleracea* var. *capitata* genotypes were found to express extremely high level stem and leaf resistances (You et al., 2016). However, when diverse sets of *Brassica* genotypes from different parts of the world were evaluated, for most genotypes there was generally no correlation between leaf and stem resistances (Uloth et al., 2013; You et al., 2016). Where there is a strong correlation, such cases share common genes (Li et al., 2015). This contrasting finding could reflect that high resistance against Sclerotinia Stem Rot in both stem and leaf is highly specific at the subpopulation level of the host species. The QTL region for stem resistance on chromosome C2 in *B. napus* was found to be homoeologous with that on chromosome A2, and falls within the syntenic region of their respective progenitor genomes (Wei et al., 2014), suggesting that the *R* genes could be inherited from a common ancestral chromosome, that is largely conserved.

Although, monogenic control of single dominant genes against *S. sclerotiorum* has been found outside the *Brassica* genus, for example in common bean (*Phaseolus vulgaris*; Schwartz et al., 2006), it is highly likely that monogenic control is present in *Brassica*s where only a HR and/or lesions ≤2 mm diameter have been observed on stems (Uloth et al., 2013, 2015a; You et al., 2016) or cotyledons (Garg et al., 2008, 2010b; Uloth et al., 2014; Ge et al., 2015). The genetic control of resistance genes is further complicated by the influence of inoculation methods (Zhao et al., 2004; Yin et al., 2010) and physiological traits, such as flowering time, as their QTL were found to be in the same genomic regions (Zhao et al., 2006; Mei et al., 2013; Wei et al., 2014). Despite this, Li et al. (2007) both defined the importance of the type and time of inoculation and how expression of field stem resistance becomes independent of flowering time provided assessment is delayed until 3 weeks after inoculation. Identification of resistance genes against *S. sclerotiorum* has been hampered by inconsistencies in time and type of inoculation. Lines that have been shown to be highly resistant in one study can be susceptible in a different study. The use of common isolates and techniques will assist in moving this research forward.

B genome *Brassica*s are commonly reported to have high levels of Sclerotinia Stem Rot resistance (Navabi et al., 2010). There is a range in resistance across the Australian *B. napus* rapeseed varieties carrying one or more *B. carinata* introgressions (Uloth et al., 2013). The Australian *B. juncea* varieties were shown to be generally more susceptible overall to Sclerotinia Stem Rot (Uloth et al., 2015a), but the Indian (Barbetti et al., 2015) and Chinese (Li et al., 2006a, 2009) *B. juncea* varieties displayed high levels of host resistance. Wild weedy Brassicaceae species have shown particular promise for high level resistance to Sclerotinia Stem Rot (Garg et al., 2010a; You et al., 2016), including genera such as *Raphanus* (Uloth et al., 2013). In particular, *B. napus* and *B. juncea* with introgression(s) from wild crucifers, including *Erucastrum cardaminoides, Diplotaxis tenuisiliqua*, and *E. abyssinicum* showed high levels of stem resistance (Garg et al., 2010a). In addition, resistance sources from the wild *B. incana* and *B. insularis* have been identified (Mei et al., 2011, 2013). Characterisation of these introgression regions using genomic technologies, and identification of candidate Sclerotinia *R* genes, through sequencing the introgression regions, will be the first step toward understanding the interaction between the host and pathogen.

The characterization of *R* genes for resistance against *S. sclerotiorum* in *B. napus* is particularly challenging. The interaction in this pathosystem is more complex than previously thought (Amselem et al., 2011). Host resistance studies in *B. napus* and other Brassicaceae genotypes have also demonstrated that not only did *S. sclerotiorum* cause pathotype-specific HR in *B. napus* cotyledons (Garg et al., 2008, 2010c; Barbetti et al., 2014), it also caused non-pathotype-specific resistance response in some genotypes of *B. napus, Raphanus sativus, B. juncea*, and *B. nigra*, where the host was resistant against different pathotypes tested (Barbetti et al., 2014; Ge et al., 2015). These two resistance outcomes against *S. sclerotiorum* rely on the specific combination between host genotype-pathogen isolate. The question is how do we distinguish these two resistances using molecular tools so that we can exploit the *R* genes? An enabler in the search for effective high level resistance to Sclerotinia Stem Rot has been the ability to define the sub-specific variation of *S. sclerotiorum* and to characterize pathotypes of this pathogen (Ge et al., 2012). This has not only provided explanation for many conflicting findings in search for effective new resistances but, more importantly, allowed identification of the first *Brassica* resistances to Sclerotinia Stem Rot that are truly pathotype-independent (Barbetti et al., 2014). Utilisation of this information and careful selection of pathotypes, inoculation methods and use of introgression lines, combined with technologies such as gene expression profiling and genome sequencing will be the first step to identification of resistance genes.

### Downy mildew

The pathogen *Hyaloperonospora parasitica* is responsible for up to 90% yield loss of Chinese cabbage (*B. rapa*) in China (Yu et al., 2009). Although, five *R* genes from *Arabidopsis* that encode both TIR- and CC-NBS-LRR have been cloned for resistance to *H. parasitica;* namely *RPP2* (Sinapidou et al., 2004) and *RPP5* (Parker et al., 1997) on chromosome 4, *RPP8* (McDowell et al., 1998), *RPP1* (Botella et al., 1998) and *RPP13* (Bittner-Eddy et al., 2000) on chromosome 3 (Botella et al., 1998), no *R* gene has been cloned from *Brassica* species. However, *Ppa3*, a single dominant *R* gene against this pathogen was mapped in *B. oleracea* using molecular markers (Singh et al., 2012). The major locus *Pp523* on chromosome C8 has also been identified to confer resistance at the adult stage in *B. oleracea* (Farinhó et al., 2004; Carlier et al., 2012), with a syntenic locus on chromosome 1 in *A. thaliana* (Farinhó et al., 2007). Another major gene *RPP31* for adult resistance against Downy Mildew was genetically mapped on chromosome 5 in *A. thaliana* (McDowell et al., 2005). Since many more *R* genes have been cloned or mapped in *Arabidopsis*, the orthologous genes in *Brassica* can be investigated through genomics approaches, for example comparative analysis of genomes (Yu et al., 2014) and pan-genome analysis (Golicz et al., 2016).

Genetic inheritance of specific resistance to *H. parasitica* has been reported in *B. oleracea* (Natti et al., 1967), *B. napus* (Lucas et al., 1988; Nashaat et al., 1997), and *B. juncea* (Nashaat and Awasthi, 1995; Nashaat et al., 2004; Chattopadhyay and Séguin-Swartz, 2005). Among these reports, Natti et al. (1967) was the only one that reported the existence of separate races (1 and 2) in broccoli (*B. oleracea* var. *italica*). Sherriff and Lucas (1990) used 33 isolates, collected from four different *Brassica* species (*B. napus, B. juncea, B. oleracea*, and *B. campestris*) and geographic locations to infect a set of *Brassica* accessions (including *B. napus, B. juncea, B. oleracea, B. carinata, B. nigra, B. campestri*s and *Raphanobrassica*). However, the study failed to delineate pathotypes of *H. parasitica*, which might explain variability in the resistance outcome. More recently, Mohammed et al. (2017) tested 131 Brassicaceae lines which highlighted excellent resistance to Downy Mildew, and was the first study to demonstrate the existence of very high levels of pathotype-independent resistance in Australian *B. napus* rapeseed varieties. However, no molecular analysis of these lines has been performed in order to identify resistance genes.

The resistance loci expressed in the cotyledon does not necessarily translate to adult plant resistance in *B. oleracea* against *H. parasitica*, as the resistance outcome can be different between the growth stages (Dickson and Petzoldt, 1993; Coelho et al., 1998). However, in some instances, there was association of resistance at cotyledon stage with that expressed at the adult stage (Jensen et al., 1999a; Wang et al., 2000). Similarly, screening tests on seedling cotyledons across *B. napus* cultivars were claimed to provide an accurate estimation of expression of field susceptibilities/resistance on seedling and more mature leaves for Downy Mildew (Ge et al., 2008). These contrasting findings indicate that the resistance outcome in the host against Downy Mildew is likely to be QTL-specific where the QTL is associated with developmental stages of the plant. Identification of such QTL should be particularly useful for breeding purposes, especially where both seedling and adult plant resistances together are required.

The genetic mechanism of resistance in the *Brassica*-*H. parasitica* pathosystem needs further clarification, as a HR has been observed in both host and non-host species (Li et al., 2011). Further, there is no evidence of clear-cut differences between *Brassica* host species which displayed a hypersensitive, partially resistant or susceptible reaction, compared with non-host species, during the early stages of infection, at spore germination and host penetration (Li et al., 2011). This may simply mean that the expression of the important components of host resistance in *Brassica*s relates to resistance mechanisms occurring during later stages of infection. Besides, *H. parasitica* infects a wide host range including *Sinapis* and *Raphanus* species and even species outside of the Brassicaceae, with these latter hosts mostly resistant to *H. parasitica* (Dickinson and Greenhalgh, 1977). Another intriguing aspect of the host resistance against *H. parasitica* is that the genetic control can be recessively inherited (Carlsson et al., 2004). Controlled infection studies using the wider host range, combined with molecular analysis such as gene expression and GWAS studies could be utilized as a first step toward understanding of the genetic mechanisms of resistance.

The sources of isolate, either geographic location from which the isolate was sourced or the host from which the isolate was found, influence the phenotypic outcome. Isolates sourced from different locations within the U.S.A. produced consistent disease scores within a test host genotype (Dickson and Petzoldt, 1993). By contrast, in Australia, significant sub-specific variation occurs and this needs to be considered in host-pathogen studies on resistance. Similarly, resistance was isolate specific whereby an English isolate was more virulent than a Danish isolate on *B. oleracea* cultivars (Jensen et al., 1999a). Unfortunately, none of these studies allows comparison of *H. parasitica* pathotype structure over time, or across different crops, different countries or continents. To date, it has not been possible to universally characterize *H. parasitica* pathotypes due to unavailability of host differentials used. Hence, there is clearly a need to develop and deploy a group of universal host differentials such that pathotypes of *H. parasitica* can be delineated, otherwise progress toward developing new varieties with resistance to the existing *H. parasitica* populations will be curtailed.

### Blackleg

The potential for *L. maculans* to cause severe damage was highlighted by the widespread total collapse of susceptible oilseed rape varieties in Western Australia in the early 1970s (Sivasithamparam et al., 2005) and subsequently in the early 2000s with the collapse of single dominant gene-based resistance from *B. rapa* ssp. *sylvestris* in various regions of Australia. *L. maculans* also causes severe damage to oilseed rape in the UK (Barnes et al., 2010), Canada and mainland Europe (Hwang et al., 2016). The less aggressive blackleg pathogen, *L. biglobosa*, is more prevalent in China (Liu Z. et al., 2014) and was recently reported to be present in New Zealand (Lob et al., 2013). Although no severe *B. napus* yield loss has been reported from *L. biglobosa*, researchers have taken precautions to prevent the spread of *L. maculans* into China (Zhang X. et al., 2014; Cai X. et al., 2017).

We focus on the host resistance against *L. maculans* in this review. Published work on sources of Blackleg resistance genes in *Brassica* species have been increasing over the years, with the focus more recently toward quantitative resistance (Jestin et al., 2011; Huang et al., 2016; Larkan et al., 2016a), and the need to bridge this historical knowledge gap has been highlighted (Delourme et al., 2006, 2012; Rimmer, 2006). Although 12 Blackleg qualitative race specific *R* genes have been genetically mapped and more have been recognized (Table 2), it remains uncertain if some of these genes are actually the same gene with different nomenclature or allelic variants of the same gene. This uncertainty arises from researchers using different crosses, different isolates and different marker systems. For example the genes *LmR1, cRLMm, Rlm4, cRLMrb*, and *LEM1* were all mapped on chromosome A7 in *B. napus* using Australian cultivars Shiralee, Maluka, Skipton and the French cultivar Major, respectively (Ferreira et al., 1995; Mayerhofer et al., 1997; Rimmer et al., 1999; Balesdent et al., 2001; Rimmer, 2006), with different molecular markers and therefore, until one is cloned, it is unknown if they are the same gene. This uncertainty has implications in breeding as breeders do not know whether they are using the same or different sources of resistance. Furthermore, it is recommended in Australia to rotate the source of resistance gene grown annually so the pathogen population cannot build up to cause severe yield loss (Van De Wouw et al., 2016b). The lack of knowledge of the number of resistance genes makes this more difficult to achieve and often means a pool of fewer resistance genes is used.

Another example relates to the *LepR3* gene in *B. napus* which has been shown to interact with *AvrLm1* (Larkan et al., 2013), however phenotypic studies suggest that *AvrLm1* may also be recognized by *Rlm1*, thus raising the question of the relationship between these two *R* genes (Rouxel and Balesdent, 2013). The relationship between *Rlm4* (Raman et al., 2012b) and *Rlm7* (Balesdent et al., 2002), implicated to be responsible for the HR outcome on *B. napus* during attack by *L. maculans* carrying the allele *AvrLm4* or *AvrLm7* respectively, is also unknown. Cloning studies on these effectors revealed that *AvrLm4* and *AvrLm7* are two distinct alleles of the same gene, termed *AvrLm4-7*, and can induce resistance in *B. napus* that harbors either *Rlm4* or *Rlm7*. However, the relationship between the two *R* genes, is yet to be determined (Parlange et al., 2009). It is also possible that *Rlm3, Rlm4* and *Rlm7* may all be allelic variations of the same gene, based on evidence showing the location of these genes on a clustered region on chromosome A7 in *B. napus* and not being found together in the same *B. napus* pure lines, with a recent study showing an unusual interplay between *AvrLm4-7* and *AvrLm3* (Delourme et al., 2004; Larkan et al., 2016b; Plissonneau et al., 2017a). Clearly there is a need to reconcile different nomenclatures for these genes and the relationship between them. Toward this, a roundtable discussion amongst experts in the field was conducted during the Brassica2016 conference and a consensus reached to join research efforts to bridge this gap (Plissonneau et al., 2017b). The success story of clarifying *AvrLmJ1* and *AvrLm5* are indeed the same *Avr* gene (Plissonneau et al., 2017a), is an example that should be followed by *Brassica* and blackleg researchers to clarify nomenclature for other genes.

Compared with other *Brassica* pathogens, the molecular characterization of avirulence genes in *L. maculans* is more widely studied (Table 3). We have now been able to study how the pathogen adapts to its new host via strict interaction at the molecular level. For example, the interaction between *Rlm3* and *AvrLm3* is suppressed by *AvrLm4-7* when attacked by the *L. maculans* isolate that carries this allele (Plissonneau et al., 2016), although this isolate can be uncommon in the Canadian rapeseed fields (Kutcher et al., 2010; Zhang H. et al., 2016). There is positive influence of the frequency of the virulent allele *avrLm1* on *avrLm6* due to genetic linkage between the two alleles in the pathogen genome (Van De Wouw et al., 2010). This observation is important when decision making is required for which variety to rotate in the field. For example, selecting a variety with *Rlm1* directly after growing a variety containing *Rlm6* would not be advised as there would be higher infection due to the presence of *avrLm1*.

**Table 3:** Additional details of in relation to *Plasmodiophora brassicae*, and *Leptosphaeria maculans*.

	***Leptosphaeria maculans***	***Plasmodiophora brassicae***
Genome size	45.12 Mbp (Rouxel et al., 2011)	25.5 Mbp (Schwelm et al., 2015)
Feature of genome	AT-rich blocks containing effector genes and transposable elements, both of which is subjected to frequent repeat-induced point mutation (RIP) (Rouxel et al., 2011)	Low number of genes, low content of repetitive elements (Schwelm et al., 2015)
Races/Isolates/Pathotypes identified and cloned (bold font has been cloned)	^*^Pathogenicity groups (Koch et al., 1991; Mengistu et al., 1991) PG1- non aggressive isolate PG2- avirulent on “Quinta” and “Glacier” PG3 – virulent on susceptible check “Westar” and Glacier but avirulent on “Quinta” PG4 – virulent on all three cultivars ^*^This PG classification was further reclassified based on different B. napus *hosts* (Badawy et al., 1991; Kutcher et al., 1993) ***AvrLm1*** (Gout et al., 2006) ***AvrLm2*** (Ghanbarnia et al., 2015) ***AvrLm3*** (Plissonneau et al., 2016) ***AvrLm4-7*** (Parlange et al., 2009) *AvrLm5* later known as ***AvrLmJ1*** (Van de Wouw et al., 2014; Plissonneau et al., 2017b) ***AvrLm6*** (Fudal et al., 2007) *AvrLm7* (Balesdent et al., 2002; Parlange et al., 2009) *AvrLm8* (Balesdent et al., 2002) *AvrLm9* (Balesdent et al., 2005) *AvrLm10* (Petit et al., 2016) ***AvrLm11*** **(Balesdent et al., 2013)** *AvrLmS* (Van de Wouw et al., 2009) *AvrLepR1* (not genetically characterized) *AvrLepR2* (not genetically characterized) *AvrLepR3* (not genetically characterized) *AvrLepR4* (not genetically characterized)	Williams differential: Race 2, 3, 4, 5, 6, 7, 8 (Williams, 1966) European Clubroot Differential: Coding system based on differential set comprising five hosts each of *B. campestris, B. napus* and *B. oleracea* using 34 European *P. brassicae* collections (Buczacki et al., 1975) Somé differential (France population): Five pathotypes, P_1_, P_2_, P_3_, P_4_, and P_5_ (Somé et al., 1996)
Affecting countries and prevalence of isolate pathotype/race found	Western Canada (2010–2011, Alberta, Saskatchewan, Manitoba): 674 isolates majority *AvrLm2, 4, 6, 7*, (S) (Liban et al., 2016) Germany (2011–2013): out of 644 isolates, majority of the tested isolates (85%) were virulent toward *B. napus Rlm* genes (*Rlm1, 3, 4, 9*). *Rlm7* remains effective. Hence, the prevalent isolates harbor: *AvrLm5, 6, 7, 8* (Winter and Koopmann, 2016) UK, Germany, Sweden, Poland (2002–2003) – 603 isolates genotyped, majority *AvrLm5, 6, 7* (Stachowiak et al., 2006) France (2000-2001) −1797 isolates genotyped, majority *AvrLm5, 6, 7, 8* (Balesdent et al., 2006) American continent (2004–2006): *AvrLm1, 2, 4, 6, 7* (Dilmaghani et al., 2009) Australia (1987–2015): *AvrLm1, 2, 3, 4, 5, 6, 7*. Frequency of *AvrLm1, 4* and *6* fluctuated across the years depending on which type of cultivar carrying specific *R* gene was commonly grown. Overall, *AvrLm4* was extremely low compared to *AvrLm1* and *AvrLm6*. Frequency of *AvrLm3* was also low (Marcroft et al., 2012a; Van de Wouw et al., 2017)	Canada (2005, Edmonton, Alberta): homogenous, ECD -/15/12 or Williams pathotype 3 or Somé P_2_ (Strelkov et al., 2007) Canada (2003, Alberta): ECD 16/15/12 and 16/15/0 or Williams pathotype 3 and 5 respectively; British Columbia ECD 16/2/12 or Williams pathotype 6; Ontario ECD 16/0/14 or Williams pathotype 6 (Strelkov et al., 2006) China: Williams race 4 (Chai et al., 2014) Czech Republic: Williams 7 most prevalent others include 6, 4, 9, 10, 2, 3, and 1 (Ríčarová et al., 2016)
Resistance breakdown	*Rlm1* (within 5 years in France) (Rouxel et al., 2003a; Sprague et al., 2006) *LepR3* (within 3 years in Australia) (Brun et al., 2000; Sprague et al., 2006), first sign of breakdown was detected within a year (Li et al., 2003) *Rlm7* (France, 6 years after commercially released 2004–2010) (Mollier, 2015). *Rlm6* (France) field trial experiment, breakdown after 3 years (Brun et al., 2010) *Rlm3* (Canada) (Zhang et al., 2016a)	“Mendel” succumbed from susceptible volunteers in Germany (Diederichsen et al., 2014)

* *This PG classification etc*.

The sources of Blackleg *R* genes were more frequently found and more diversified in the winter type *B. napus* compared to the spring-type cultivars (Rouxel et al., 2003b), where *Rlm4* is the most common *R* gene in the latter (Rouxel et al., 2003b; Marcroft et al., 2012a). Both *B. rapa* and *B. napus* have proven to be good sources of Blackleg resistance genes on the A genome. Some genes from *B. rapa* have been introgressed into *B. napus*, such as the *LepR*-series of *R* genes from *B. rapa* ssp. *sylvestris* (Yu et al., 2005, 2008, 2013). Researchers have also attempted to exploit the B genome for resistance, and *R* genes/QTL that are highly resistant to *L. maculans* have been introgressed from the B genome (Rimmer and van den Berg, 1992; Plieske et al., 1998), particularly into *B. napus* (Sacristan and Gerdemann, 1986; Chevre et al., 1996; Fredua-Agyeman et al., 2014). The resistance level in the introgressed *B. napus* was the same as that in the donor B genome source, although expression of phenotype was shown to be influenced by temperature (Plieske et al., 1998). These novel sources of *R* genes introgressed into *B. napus* will enable continued resistance against the pathogen, through expanding the number of resistance alleles available for breeding, particularly in times of climatic uncertainty.

Both *LepR1* and *LepR2* are involved in cotyledon resistance and are also associated with resistance at the adult stage in *B. napus* (Yu et al., 2005). However, the mechanism of resistance at both stages is different because Yu et al. (2005) showed that presence of *LepR1* provided complete resistance, while *LepR2* only offered partial resistance at the cotyledon stage, yet both genes ensured complete resistance at the adult stage. A very different pattern of resistance exists for the Chinese *B. napus* lines derived from winter type and spring type parents, where *R* gene mediated resistance in these lines conferred by both *Rlm3* and *Rlm4* was effective at both seedling and adult plant stages against *L. maculans* (Zhang et al., 2016b). Similarly, the Australian winter *B. napus* lines harboring either *Rlm1, Rlm3* or a combination of both display stable resistance against *L. maculans* where resistance is effective at both seedling and adult plant stages (Light et al., 2011). Such examples, taken together, imply that while *R* genes can be effective at both cotyledon and adult stages, they can also act interdependently or dependently at different plant developmental stages, and are likely affected by the genotype of host and pathogen, as well as by environmental conditions. The *R* gene effective at seedling stage can also lie in the same QTL region as that of adult plant resistance (Raman et al., 2012b). Whether this adult plant resistance is mediated by the same *R* gene or by quantitative non-specific resistance genes is yet to be confirmed. An increased understanding of this molecular basis for resistance, for example through further genome sequencing and gene cloning, will enable the genes to be deployed more effectively. Further, expression of *R* gene mediated resistance can also be expressed differently across other host plant components; for example, *Rlm1* and *Rlm4* exhibit major gene resistance on *B. napus* pods, not only on cotyledons (Elliott et al., 2016).

Besides qualitative resistance, quantitative resistance has also been assessed and reported in *B. napus* against *L. maculans* (Huang et al., 2009). Methods to determine quantitative resistance more efficiently such as inoculation at different parts of the leaves under controlled conditions have been evaluated (Huang et al., 2014). Several QTL responsible for quantitative resistance against *L. maculans* have been identified in French, European and Australian *B. napus* cultivars (Jestin et al., 2011; Raman et al., 2012b; Huang et al., 2016; Larkan et al., 2016a). However, the genetic control of quantitative resistance against Blackleg is poorly understood as it is very difficult to compare QTL, even with the same molecular markers and lines being utilized, due to environmental differences and a lack of knowledge of the pathogen presence in the field.

## Genetics

### *R* genes/QTL mapped

The genetic inheritance of pathogen resistance, based either on a gene-for-gene interaction where the *R* gene is either a single dominant gene (complete resistance) or involves multiple genes (partial resistance) has been established for the four key pathogens of this review (Table 2). The *R* genes/QTL mapped for the key pathogens are illustrated in Figure 1.

Whilst numerous QTL and *R* genes have been genetically mapped (Table 2), to date, only four resistance genes have been cloned, for Clubroot and Blackleg resistance. Two Blackleg resistance genes have been cloned; *LepR3* was cloned from *B. napus* cv. Surpass 400 (hybridization between *B. rapa* ssp. *sylvestris* and *B. oleracea* ssp. *alboglabra*) (Larkan et al., 2013) and *Rlm2*, an allelic variant of *LepR3*, from Glacier DH24287 (Larkan et al., 2015). *LepR3* is characterized to have a similar function to the *Cf-9* gene from tomato that confers resistance toward the biotrophic ascomycete fungus *Cladosporium fulvum* (Jones et al., 1994), which shares the same mode of attack as *L. maculans* (Stotz et al., 2014). *LepR3/Rlm2* are receptor-like proteins (RLP) that interact specifically with the effectors *AvrLm1* and *AvrLm2*, respectively, at the plant cell surface. This class of disease resistance protein is not the same as the cloned *R* genes for *P. brassicae* in *B. rapa* which are NBS-LRRs, thus raising the question, is RLP considered part of effector triggered immunity (ETI)? In recognizing this “gray area,” a review by Stotz et al. (2014) tried to re-classify plant immunity for apoplastic fungal pathogens such as *L. maculans* and *Cladosporium fulvum*, for which the defense mechanism seemed to deviate from the classical NBS-LRR recognition in the host cell cytoplasm where effectors are being secreted. Stotz et al. (2014) proposed the new resistance defense triggered by apoplastic fungal pathogens as “effector-triggered defense” (ETD) that involves RLPs recognizing effectors outside host cell cytoplasm. In *Brassica* species genome wide *R* gene identification studies have focused only on NBS-LRR genes, future studies should include RLPs and RLKs in the analysis, as this may pave the way for cloning of further *R* genes in *Brassica* species (Rameneni et al., 2015; Sekhwal et al., 2015; Li P. et al., 2016).

Recent advances in genome sequencing and assembly have allowed us to tap into the rich genetic resources from different crop species and their wild relatives (Bevan et al., 2017). A reference genome is useful to align and compare all the different QTL mapping experiments. By having a reference genome, the physical location of the markers in QTL maps that are linked with candidate genes can be determined (Zhang Y.-M. et al., 2016). Currently, the genome sequence of the *Brassica* diploids, *B. rapa* (Wang et al., 2011; Cai C. et al., 2017), *B. oleracea* (Parkin et al., 2014; Liu S. et al., 2014), and *B. nigra* (Yang et al., 2016) and the amphidiploids *B. napus* (Chalhoub et al., 2014; Bayer et al., 2017), *B. juncea* (Yang et al., 2016) have been published. This along with the advanced genotyping tools such as the 60 K Brassica Infinium SNP array (Clarke et al., 2016) and Genotyping by Sequencing (GBS) (Bus et al., 2012; Bayer et al., 2015; Cheng et al., 2016; Scheben et al., 2017a), have allowed rapid identification of genome-wide QTL and GWAS regions for *R* genes for Clubroot (Lee et al., 2016; Li L. et al., 2016), Sclerotinia Stem Rot (Wei L. et al., 2016; Wu et al., 2016a) and Blackleg (Raman H. et al., 2016).

**Figure 2:**
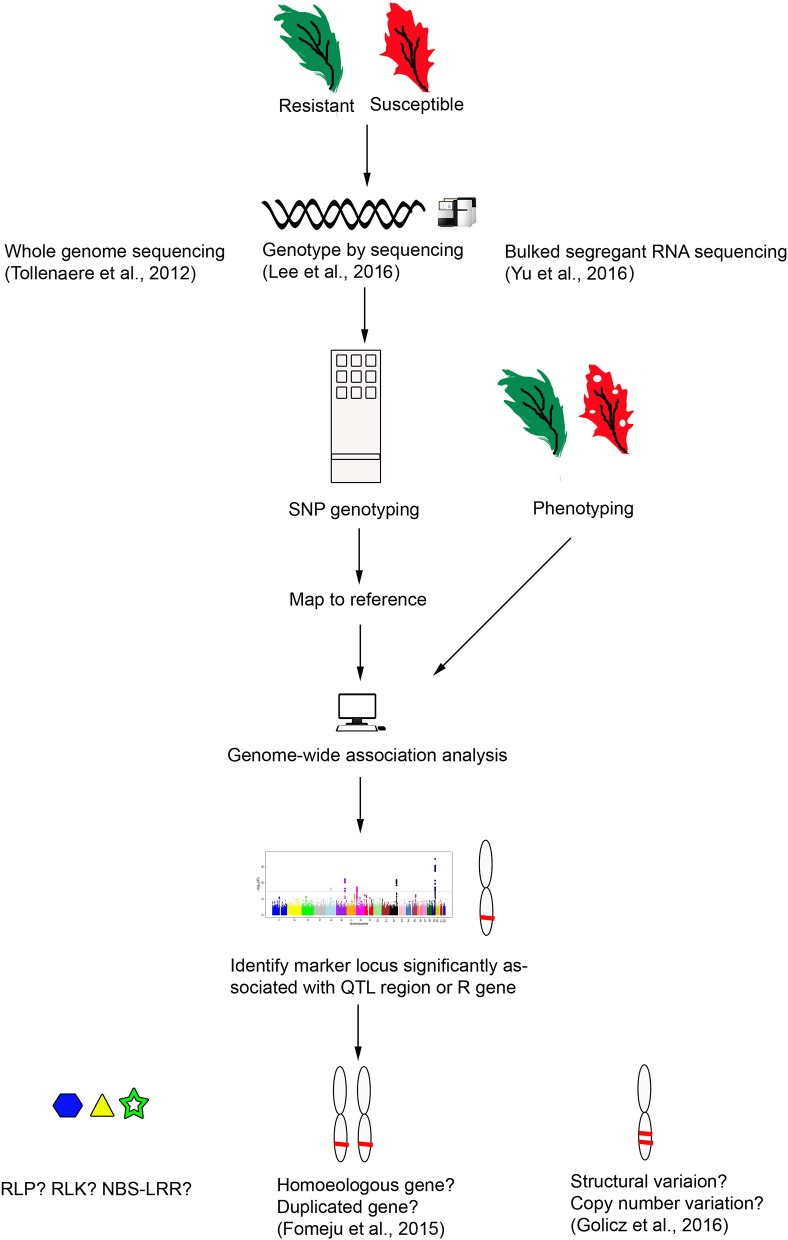
Identification of *R* genes in *B. napus* using next generation sequencing methods.

Genome sequencing in *Brassicas* has now been expanded to include the *B. oleracea* pan-genome where genomes of nine lines were assembled and compared for structural variation (Golicz et al., 2016). Pan-genome sequencing is an important advance, as the diversity of a species is not represented by a single cultivar, and in *B. oleracea* only 81% genes were present in all lines. Of the 19% variable genes, those associated with disease resistance were found to be among the most prevalent. Therefore, in identification of resistance genes we should look at the variable genes, not only those in the reference genome sequence. Genome resequencing (Cheng et al., 2016) and pan-genomics (Golicz et al., 2016) approaches are promising tools to make identification, characterization and cloning of candidate *R* genes more achievable and reliable (Figure 2). The pan-genome approach to study structural variation of the genes in *Brassica* genomes, with whole-genome gene expression and methylation studies has already proved very useful toward uncovering the structure, function and evolutionary origin of *R* genes (Parkin et al., 2014; Golicz et al., 2016). Copy number variation (CNV) has already been studied for the AP2/ERF superfamily involved in plant stress tolerance in *B. napus*, CNV of *R* genes in *Brassica* is only now being investigated (Batley et al., 2016) and has been implicated in important crops such as cereals (maize and rice), Solanaceae and soybean (Springer et al., 2009; McHale et al., 2012; Xu et al., 2012; Saxena et al., 2014; Wei C. et al., 2016). This gene expansion through structural variation allows the plant to adapt to rapid changes in the environment (Cheung et al., 2009).

## Challenges

### Genome complexity

*Brassica napus* is a relatively young crop in evolutionary history that results from an accumulated series of polyploidisation events (Mason and Snowdon, 2016). Prior to hybridization, the whole genome triplication in the diploids (*B. rapa* and *B. oleracea*) was preceded by three events of whole genome duplication in the ancestor genome that have further contributed to complicated gene rearrangement, duplication and loss (Town et al., 2006; Chen, 2007). Despite the genome complexity, *Brassica* species have become models to study evolutionary events and how rich genetic resources can be exploited from the plant (Pires and Gaeta, 2011; Liu S. et al., 2014).

Domesticated *B. napus* has a lower genetic diversity compared to its diploid progenitors *B. rapa* and *B. oleracea*, evidenced by similar gene order and content between the three species due to strong genetic bottlenecks during cultivation (Cheung et al., 2009; Mason and Snowdon, 2016). By contrast, homoeologous rearrangements/recombination occurs more frequently in resynthesized *B. napus*, thereby contributing greatly toward genetic diversity, which provides a good source of *R* genes for breeding resistant *B. napus* cultivars (Gaeta and Pires, 2010). Recently, efforts were made to produce introgressed *B. napus* in a novel way, from *B. juncea* (A genome source) and *B. carinata* (C genome source) (Chatterjee et al., 2016) (rather than from *B. oleracea* and *B. rapa*) from where more sources of *R* genes can then be utilized and identified. While in *B. napus*, the homoeologous genes are often co-expressed (Chen, 2007), there could also be bias in homoeolog expression, as found in *B. juncea* (Yang et al., 2016) and *B. rapa* (Cheng et al., 2012). How the different levels of gene expression affect the stability of *R* genes remains to be determined. The fate of homoeologous genes, whether they are silenced, expressed, or both copies diverge where the gene function may or may not change (sub- and neo-functionalization) is a result of the genetic and epigenetic interactions between redundant genes (Comai, 2005), biased gene expression (Yang et al., 2016), gene fractionation (Subramaniam et al., 2013) or accelerated amino acid sequence evolution and positive selection (Liu and Adams, 2010). These polyploidy events could potentially result in a selective advantage, for example higher seed yield in *B. napus* (Osborn et al., 2003).

Although race-specific *R* genes are easier to manipulate for breeding resistant crops compared to polygenic resistance, which involves many genes and the effect of environmental factors, the identification of *R* genes in *B. napus* still remains a challenge due to the complexity of its genome. For example, a major QTL for resistance to *S. sclerotiorum* was duplicated through homoeologous non-reciprocal transposition (HNRT) in *B. napus* (Zhao et al., 2006). The quantitative resistance genes against *L. maculans* are affected by gene duplication in homoeologous regions in *B. napus* (Fomeju et al., 2014, 2015). *L. maculans R* genes, *Lmr1* and *ClmR1* from cultivars Shiralee and Cresor respectively, were mapped on regions that are highly duplicated within and between genomes in *B. napus*, and originated from the common ancestor before divergence of *B. oleracea* and *B. rapa* (Mayerhofer et al., 2005). Evolutionary information regarding resistance genes such as these is useful for breeding purposes, as it highlights specific genera/species likely to be most useful in developing new cultivars with highly effective and durable disease resistances. For *H. parasitica*, the single dominant gene *Pp523* was mapped onto two regions on chromosome C8 and another on C5, reflecting triplicated regions in *B. oleracea* (Carlier et al., 2011).

The success stories of cloning *Crr1a* and *CRa* (Clubroot *R* genes encoding TIR-NB-LRR) from *B. rapa* (Hatakeyama et al., 2013) and *LepR3*/*Rlm2* (Larkan et al., 2013) in *B. napus*, revealed that the *R* genes are organized as members of allelic variants or tandem repeats. The clustering effect of the *R* genes for these two pathogens provides further evidence of complexity of the genome organization. Clustering of *R* genes is also prevalent in association with Clubroot, Blackleg, and Sclerotinia Stem Rot resistance on both *B. rapa* (Kato et al., 2013) and *B. napus* (Delourme et al., 2004; Kato et al., 2013; Li et al., 2015). Such gene clustering effects are common in plant genomes for quick adaptive response to pathogens via recombination events (Hulbert et al., 2001; Meyers et al., 2003). Clustering of *R* genes may potentially enable recognition of different pathogen races carrying different avirulence genes, such as in Clubroot *R* genes on chromosome A8 (Pang et al., 2014). Epistatic interaction was also observed for Clubroot in *B. napus* (Manzanares-Dauleux et al., 2000), where a single dominant resistance gene does not explain the resistance outcome in its entirety. It was also reported that the same genomic region in *B. napus* can act as both QTL and/or major gene based on different pathotypes of Clubroot (Manzanares-Dauleux et al., 2000; Rocherieux et al., 2004). The clustering and duplication of *R* genes can also cause problems in *R* gene identification as these genes tend to collapse in genome sequence assemblies. Reliance on the single reference genome sequence can therefore hamper efforts and future work should incorporate novel technologies such as isolated chromosome sequencing or targeted amplification of genomic regions of interest.

### Genetic variation

Further to the complexity of *R* genes, resistance levels vary with different host species within *B. napus* for the key pathogens discussed in this review. Often, intermediate/heterogeneous phenotypes were obtained when using the pathogen isolates either derived from differential sets or from host cultivars. This could be due to genetic variation within the host cultivars and/or even within the pathogen.

#### Host

*Brassica napus* has a low genetic diversity due to the recent evolutionary history (Snowdon et al., 2007; Prakash et al., 2011). Although *B. napus* has a comparatively lower level of genetic variation (Wu et al., 2014), breeding programs have made extensive use of interspecific hybridization within the Brassicaceae family, including with *Sinapis arvensis*, to broaden the genetic diversity for Blackleg resistance (Winter et al., 2003; Snowdon et al., 2007). Depending on the genetic background of the *B. napus* host, for example winter, spring type cultivars or resynthesized lines, the *R* gene diversity can be high or low (Rouxel et al., 2003b). This can affect the assessment of disease on the plant where a wide range of scores is obtained (Larkan et al., 2016b).

Genetic mapping studies on the key pathogenic resistances showed that breeding materials or cultivars were mostly used to generate mapping populations (Table 1; Diers and Osborn, 1994; Plieske and Struss, 2001). However, other genetic sources should be tapped into to increase the genetic pool for breeding resistant *B. napus*. For example, resynthesized lines can provide a good source of genetic diversity compared to cultivars (Becker et al., 1995). This is evidenced by the successful introgression of the resistance gene *LepR3* from *B. rapa* into *B. napus* (Larkan et al., 2013). Fodder and vegetable rape genetic material can also contribute significant amount of *R* genes, because of high genetic variation in these species (Hasan et al., 2006). Genome-wide SNPs have been studied in natural accessions and synthetic *B. napus*, inferring sequence polymorphism in genes linked to agronomically important traits such as seed quality (Huang et al., 2013; Qian et al., 2014; Schmutzer et al., 2015) and this could be expanded to disease resistance studies.

#### Pathogen

A high level of genetic variation in *S. sclerotiorum* populations was found in *B. napus* fields in Australia (Sexton et al., 2006) and Canada (Kohn et al., 1991). In particular, the Australian *S. sclerotiorum*, with different isolates/pathotypes, caused variable resistance outcomes ranging from intermediate resistance to a HR to complete resistance in different host *B. napus* genotypes (Garg et al., 2010b; Ge et al., 2015). This poses important questions regarding this pathosystem: Does this host resistance come from specificity of the host species genotype, from the pathogen genotype or from a combination of both? Is there a role for non-host resistance to adapted pathogens? Could there be specificity of the host cultivar? Can host specificity and non-host specificity be displayed in different genotypes of the same host? Until the genetic basis for the various resistance levels in this pathosystem is unveiled, we will not know if the *B. napus* genotypes are going through a process of losing or acquiring host status (marginal host), or are intermediate between host and non-host status, much like the barley resistance to rust where the host-pathogen interaction is indeed very specialized (Niks and Marcel, 2009).

For *L. maculans*, high genetic diversity has been found within populations. In France, evidence for this was found through an isolation by distance (IBD) study where there was high gene flow and high dispersal rates of spores among populations across large geographical distances (Travadon et al., 2011). These characteristics offer the pathogen a survival advantage to cope with the host resistance in the field, highlighting the role of co-evolutionary dynamics between pathogen and host in conferring resistance. Similarly, in Australia, *L. maculans* also displayed high genetic variation between geographic locations such that the isolates from Western Australia were shown to be genotypically different from those collected from the eastern states. Studies using SNPs (Zander et al., 2013; Patel et al., 2015), microsatellite and minisatellite markers (Hayden et al., 2007) indicated that some populations are panmictic. Gene loss in *L. maculans* has contributed to genetic variation between isolates (Golicz et al., 2015). The source of genetic diversity for *L. maculans* could also depend on the specific host morphotype from which the pathogen is adapted to. For instance *L. maculans* “*brassicae”* from cultivated *Brassica* and *L. maculans* “*lepidii*” from *Lepidium* sp. were identified in the International Blackleg of Crucifers Network (IBCN) collection as one of the seven subgroups of the species complex based on ITS-RFLPs (Mendes-Pereira et al., 2003). This species complex, differing in specificity and pathogenicity on *B. napus*, potentially complicates the cloning of *R* genes in *B. napus* (Voigt et al., 2005).

High genetic diversity was reported for *P. brassicae*. For example, in Japan where the pathogen showed different pathogenicity levels on the cruciferous crops and cruciferous weeds (Jones et al., 1982; Tanaka and Ito, 2013). However, pure pathotype/genotype isolates of *P. brassicae* can still be obtained from single root hairs, which minimizes the variability of pathogenicity, thus a more reliable interpretation of the host-pathogen relationship can be established (Diederichsen et al., 2016). For *H. parasitica*, a molecular study also revealed high genetic diversity within the species, probably due to the broad host range of this pathogen where it can infect, for example, various *Brassica* and *Raphanus* species (Choi et al., 2003).

### Pathotype classification

With the exception of the recent development of host differentials to delineate pathotypes of Sclerotinia Stem Rot (Ge et al., 2012), host differential sets to delineate pathotypes/races of the other pathogens have generally been established for some time (as early as the 1960s). Examples include Clubroot (Williams, 1966; Buczacki et al., 1975; Somé et al., 1996), Blackleg (Mengistu et al., 1991; Purwantara et al., 2000; Balesdent et al., 2002, 2005; Van de Wouw et al., 2009) and Downy Mildew differentials (Natti, 1958). The usefulness of pathotype classification became obscured when research groups used unknown, different and/or universally unobtainable sources of host differentials that prevented application of a universal and consistent set of host differentials. The “loss” of the Downy Mildew differentials used by Natti (1958) well illustrates these challenges. Application of different host genotype differentials not commonly available across different locations and/or countries has meant findings in terms of definition of pathogen races/pathotypes is, at best, of limited use and then only to that particular locality. Use of pathogen isolates without appropriate pathotype/race classification, can easily lead to misleading *R* gene identification when it comes to qualitative or quantitative resistance classifications. To overcome this, an additional differential host differential set was established for Clubroot in Japan using local Chinese cabbage cultivars and local field isolates (Kuginuki et al., 1999; Hatakayema et al., 2006) and similarly for Downy Mildew using European isolates from *B. oleracea* (Coelho et al., 2012). Furthermore, as new pathogen isolates carrying new avirulence genes emerge as a normal consequence of pathogen evolutionary dynamics, host differential sets need to be updated and standardized to enable them to effectively delineate future new pathotypes/races.

Application of different scoring systems could also lead to complications in the identification of *R* genes. For example, in Downy Mildew, at least three scoring systems have been applied (Dickson and Petzoldt, 1993; Wang et al., 2000). It is also imperative that a robust race/pathotype differentiation system be utilized to separate different and group similar races/pathotypes. While for *L. maculans* and *P. brassicae* established race differentiation systems are robust, for *H. parasitica* and some other pathogens this is not the case. A little utilized but extremely robust system was developed and utilized for the barley scald fungus, *Rhynchosporium secalis* by Goodwin et al. (1990). The Goodwin et al. (1990) pathogen isolate differentiation system utilizes an octal nomenclature classification that codes pathogen isolates according to their pathogenicity (expressed as disease severity) on the host differentials, mathematically confirming appropriateness of differential host and then grouping these genotypes into sets according to their genetic inheritance. This allows continuity with any previous studies that have different nomenclatures. Its suitability is illustrated by its recent successful application to define the first pathotypes within *S. sclerotiorum* populations on *B. napus* within Western Australia (Ge et al., 2012). Despite these differentials being made available worldwide, upon request, there has been little uptake outside of Australia, India and Eastern Europe to date. The Goodwin et al. (1990) pathogen isolate differentiation system applied to *H. parasitica* isolates would also be highly beneficial for the same reasons.

### Rapid evolution of virulent pathotypes and host-pathogen interaction

In *B. napus*, resistance toward race-specific pathogens such as *L. maculans* and *P. brassicae* has been overcome. New virulent pathotypes of *P. brassicae* that have the potential to cause resistance breakdown in *B. napus* have been identified (Strelkov et al., 2016; see Table 3). Mass cultivation of cultivars containing particular *R* genes favors selection of pathogen strains that are better adapted or have greater virulence due to strong selection pressures exerted on the pathogen. Care should be taken that introgressed lines with single dominant gene resistance are not widely grown homogeneously in commercial fields year after year (Marcroft et al., 2012b). Whilst they may be effective in the short term (one to two seasons), they will likely lose their efficacy over a period of time, as occurred in as little as 3–4 years for *L. maculans* (Li et al., 2003). In France, complete deletion of the Blackleg avirulence gene *AvrLm4-7* was found (Daverdin et al., 2012).

The genetic mechanism of resistance in *Brassica* is different for each pathosystem. For the *B. napus*-*L. maculans* and -*P. brassicae* pathosystems, it is generally straightforward because distinct major gene resistances and respective cotyledon and root phenotypes have been well established using the Blackleg differential and Williams (1966) systems, making host single dominant *R* gene effects relatively easy to study. In contrast, in the *B. napus*-*S. sclerotiorum* pathosystem, although a hypersensitive reaction was found for *S. sclerotiorum* on cotyledons (Garg et al., 2008; Ge et al., 2015) and stems (Garg et al., 2010a), it occurred in relation with specific host genotype-*S. sclerotiorum* isolate or pathotype combinations. In other words, the resistance outcome is pathotype-dependent and genotype expression of resistance is tied to the selected isolate or pathotype (Barbetti et al., 2014). Further, any QTL or gene expression results are tied to these particular host genotype-*S. sclerotiorum* isolate or pathotype combinations. The fact that seedling vs. adult and leaf vs. stem resistances are generally independently expressed (Uloth et al., 2013, 2014), has inhibited advancement of our understanding at a molecular level in comparison with that for *L. maculans*. Initially, for *S. sclerotiorum*, until there is wider utilization of the differentials developed to distinguish distinct pathotypes of *S. sclerotiorum*, as developed by Ge et al. (2012), it may be productive for molecular studies to focus more on using pathotype-independent resistances (i.e., genotypes showing resistance to all pathotypes) as recently highlighted by Barbetti et al. (2014). Failure to define sub-specific variation in *S. sclerotiorum* remains an impediment to rapid progress in our understanding at a molecular level with the host genotype-*S. sclerotiorum* pathosystem. Further, it is evident that gene expression within the pathosystem involving *S. sclerotiorum* can be differentially expressed across different host genotypes depending upon their sensitivity toward environmental effects, for example small changes in temperature (Uloth et al., 2015b). Such variation further challenges outcomes for molecular studies into resistance against this pathogen, particularly with variations in expression of resistance across different temperatures, especially when temperature-phenotypes occur (Uloth et al., 2015b). Similar challenges occur in relation to the host genotype-*H. parasitica* pathosystem and likely occur wherever sub-specific pathogen variation occurs but has not been definitively characterized. In addition, comparisons made with known *S. sclerotiorum* pathotype delineations across approximately 60 Western Australian isolates showed that there was no relationship between pathotype with microsatellite or with IGS haplotype (Clarkson et al., 2017). Clearly there is a need for further molecular work, for instance metabolomics and proteomics, in addition to genomics, in conjunction with appropriate phenotyping across a range of environmental conditions to further investigate and understand these challenges both now and for the future.

### Abiotic conditions

Climatic conditions can influence the interaction between plants and pathogens and the pathogen distribution, contributing directly to the disease outcome on the host (Barbetti et al., 2012). Abiotic conditions including temperature and soil nutrient content could influence the resistance spectrum on the host *A. thaliana* against powdery mildew pathogens (Jorgensen, 2012). In the *L. maculans*-*B. napus* pathosystem, temperature effect influences the phenotypic outcome at different plant growth stages. For example, in general, during infection at nearly all growth stages from cotyledon to pod at higher temperature regimes (e.g., 18/24°C), resistance expressed at the seedling stage was positively correlated with the adult stage, as compared to lower temperature regimes (e.g., 11/18°C) (Li et al., 2006b). Warmer temperature and high humidity levels favored *L. maculans* colonization of the host during a *Rlm6*-mediated resistance study on *B. napus* (Huang et al., 2006), confirming a related study on *Rlm1*-mediated resistance by Badawy et al. (1992). Cold temperature and low soil acidity slow down or inhibit *P. brassicae* infection on *B. napus*, while the influence from soil micronutrient stops at high inoculum concentration (Gossen et al., 2014).

## Strategies

### Novel sources of *R* genes and transfer of *R* genes

Novel sources of *R* genes for Blackleg can be sourced from other A- and C-genome *Brassica* species (Chen et al., 2010) or from the B genomes in *B. nigra* (Chevre et al., 1996), *B. juncea* (Christianson et al., 2006), and *B. carinata* (Plieske et al., 1998). Wild Brassicaceae species, such as *Sinapis arvensis*, also serve as a good source of *R* genes for Blackleg (Snowdon et al., 2000). However *R* genes from other wild species remain to be tapped. Besides identifying candidate *R* genes through comparative genomic analysis, more screening work on wild and land races, including species from the Brassicaceae family, should be performed against the main pathogens to identify resistant plants. This would broaden the gene pool for introgression of novel *R* genes or alleles into *Brassica* cultivars. Combining this information with genome sequencing will enable characterization of these novel resistance genes.

Although effector genes are usually unique and do not often share structural similarity but encode very diverse proteins, it is still possible to identify an *R* gene that could recognize common or near identical effector gene(s) in different pathogen pathotypes/races/isolates, which already exists naturally in some plants/crops (Dangl et al., 1992). This type of *R* gene is useful and can be transferred between hosts that are closely related or within the same genus or family. Some *R* genes can also be transferred from host to non-host using GM technologies, providing broad-spectrum immunity against one or more pathogens. One such successful example is the pepper *Bs2* gene against bacterial spot disease transferred from its host pepper into non-host tomato (both members of Solanaceae family; Tai et al., 1999). The *Bs2* gene encodes the *R* gene NBS-LRR class (Tai et al., 1999) while the gene *avrBs2* in *Xanthomonas campestris* pathovar *vesicatoria*, causal pathogen of bacterial spot disease, is essential for virulence activity and is highly conserved between and within the pathovars of *X. campestris* (Kearney and Staskawicz, 1990). This has opened up new possibilities to protect economically valuable crops, that lack *R* gene resources, from disastrous pathogens.

### Breeding resistant cultivars

Continuous efforts are on-going to breed resistant *B. napus* cultivars through introgression. *B. rapa* remains a good source of race specific *R* genes that often result in high resistance against Clubroot (Strelkov et al., 2006) and Blackleg (Yu et al., 2008). For Clubroot, a single dominant gene from resistant *B. rapa* turnip morphotypes was successfully introduced into Chinese cabbage (Yoshikawa, 1980). Transfer of a Clubroot resistance gene was also performed from *B. napus* into *B. oleracea*, with the resistance gene believed to also be carried in the A genome of *B. rapa* (Chiang et al., 1980). In Canada, efforts to transfer a Clubroot resistance gene from the *B. napus* hybrid “Mendel” into a local susceptible spring *B. napus* cultivar was promising (Rahman et al., 2011). Transfer of a Clubroot *R* gene was also made from *B. rapa* Chinese cabbage “Qulihuang” into *B. napus* “Topas” using marker-assisted selection (Hirani et al., 2016). For *L. maculans, R* genes have been genetically mapped and identified in the A genome of *B. napus* (Alamery et al., in press). For *S. sclerotiorum*, introgression lines of *B. juncea* carrying genomic segments from the wild Brassicaceae *B. fruticulosa* demonstrated marker trait association for Sclerotinia Stem Rot resistance (Rana et al., 2017). Within breeding programs, it is important to make informed decisions about the source of novel *R* genes and which parental genomes to cross, because the *R* gene from the parents may be masked in the progenies of the resynthesized genomes due to one or more epistatic interactions, such as was found for a Clubroot *R* gene (Werner et al., 2007). These challenges can be overcome by the latest genome editing techniques, such as CRISPR-Cas9 (Scheben et al., 2017b), as was recently reported in maize with precise mutation on the targeted allele (Svitashev et al., 2016; Liang et al., 2017).

The B genome offers good sources of resistance against two other pathogens besides *P. brassicae* and *S. sclerotiorum*; White Rust and White Leaf Spot (*A. candida* and *P. capsellae*, respectively). For White Rust, potential sources of resistance genes come from *B. nigra* and *B. juncea* (Westman et al., 1999), while resistance against White Leaf Spot potentially comes from *B. carinata* (Gunasinghe et al., 2016). For White Leaf Spot, in contrast with Clubroot and Blackleg, *B. rapa* is more susceptible than *B. napus* (Gunasinghe et al., 2014), but as with Downy Mildew, there is good resistance to White Leaf Spot in *B. oleracea*. In fact, complete resistance was recorded in *B. oleracea* var. *capitata* (Gunasinghe et al., 2016). However, *B. juncea* originating in India or Australia is highly susceptible to White Leaf Spot (Gunasinghe et al., 2014).

To breed resistant cultivars, it is important to consider the fitness cost to pathogen isolates when they do overcome host resistances. We can study the pathogen avirulence/virulence genes in the field that are adapted to the host cultivar and determine the genetic changes in the pathotype that cause heavy fitness consequences (Vera Cruz et al., 2000; Delmas et al., 2016). It is also important to consider the host *R* gene as selection pressure on the pathogen can affect the stability of the *R* gene. In terms of the linked genes in *L. maculans, AvrLm1*, and *AvrLm6*, high selection pressure on one *Avr* gene can cause a change in allele frequency of the other (Brun et al., 2010; Van De Wouw et al., 2010). Understanding the evolutionary potential between the pathogen and the host is essential in developing a durable resistant *Brassica* crop (McDonald and Linde, 2002).

### Co-infection of *R* genes to immunise *Brassica* crops

In the *L. maculans*-*B. napus* cultivar “Surpass 400” pathosystem, mixed inoculation of isolates (avirulent and virulent) with contrasting phenotypic effect (resistant vs. susceptible), can render *B. napus* resistant, even with only a small volume of avirulent isolate in the mixture of the two isolates (Li et al., 2006a). Such “protection” effects are also observed in the field in Australia and have also been observed in the *Phytophthora megasperma* f.sp. *glycinea*–soybean pathosystem where a mixed isolate inoculation controlled the lesion spread (lower rate of spread; Ward, 1983). While the mechanism of improved resistance involving mixed isolate inoculations remains unknown, this approach does offer potential for possible “immunization” of host plant and further investigations into such are warranted. However, it should also be noted that the reverse can happen where co-infection results in increased disease susceptibility of the host. For example, co-infection of different pathogens such as *A. candida* (causal agent for White Rust) and *H. parasitica* (Downy Mildew) where the two pathogens can grow together within leaf and/or stem tissues of the same host plants (Cooper et al., 2008). Cooper et al. (2008) also showed that pre-infection of virulent *A. candida* can influence an otherwise avirulence outcome for *H. parasitica* such that it became virulent in *B. juncea*, as was also shown by Singh et al. (2002). However, pre-inoculation of the virulent *H. parasitica* on White Rust-susceptible *B. juncea* host promotes better resistance to White Rust disease (Singh et al., 2002), suggesting that type of isolate, infection sequence and pathogenicity of the isolate could determine the resistance outcome in the host (Kaur et al., 2011). The molecular basis of these co-infections across multiple pathogens remains unknown.

### Defense-related genes other than *R* genes

In addition to the up-regulation of HR-related genes characterized by signaling hormones (Chu et al., 2014; Nováková et al., 2016b; Uloth et al., 2016), regulatory genes such as the transcription factors WRKY (Zhou et al., 1997) and calmodulin-binding transcription activator (CAMTA) (Rahman H. et al., 2016) have also been implicated to regulate the disease response in *Brassica* pathosystems (Zhou et al., 1997). The WRKY gene was studied in *B. napus*-*S. sclerotiorum* and -*Alternaria brassicae* pathosystems, where gene expression was increased upon pathogen infection alongside defense-related hormones (Yang et al., 2009; Zhao et al., 2009). This gene was shown to enhance resistance against Downy Mildew in the Chinese broccoli *B. oleracea* var. *italica* (Jiang et al., 2016). Besides the WRKY gene, for the same host species, a pathogenicity-related defensin gene, which encodes defense-related cysteine-rich protein has also been shown to improve resistance against Downy Mildew (Jiang et al., 2012) and *S. sclerotiorum* (Zarinpanjeh et al., 2016). Identification of candidate genes for Clubroot resistance revealed Rho proteins may be involved (Kato et al., 2013). In Head Smut in maize, *ZmWAK* was successfully identified as a regulator gene with quantitative effects on host resistance expression (Balint-Kurti and Holland, 2015; Zuo et al., 2015). Further characterization of these genes should be performed in *Brassica* species, through genome wide identification and gene expression studies to determine their role in disease resistance.

### Molecular identification of pathotype and pathogen elicitors/effectors

To control disease outbreaks in crops, it is imperative that pathogen population variability in the field can be quickly identified. This can be achieved by having a full set of genetic markers for different pathogen pathotypes, not only contained within the local fields but universally. For example, initiatives for *P. brassicae* showed that *Cr811* was a good candidate gene to differentiate pathotype 5 from the rest of the pathotypes (Feng et al., 2016).

Molecular characterization of pathogen effectors is gaining momentum. With better genome assemblies and higher gene prediction accuracies, we will soon have better insights into the structures and functions of effector proteins, which allows more efficient screening and identification of *R* genes (Gibriel et al., 2016). Elicitor candidates for *L. maculans* (Wilson and Howlett, 2005; Nováková et al., 2016a), and effector candidates for *S. sclerotiorum* (Guyon et al., 2014), are both responsible for inducing HR. RNAi studies established that the transcription factor LmStuA in *L. maculans* regulates expression of effector genes (Soyer et al., 2015). Genomic studies researching *L. maculans* effectors *AvrLmJ1* (Van de Wouw et al., 2014), *AvrLm1* (Gout et al., 2006), *AvrLm2* (Ghanbarnia et al., 2015), *AvrLm3* (Plissonneau et al., 2016), *AvrLm4-7* (Parlange et al., 2009), *AvrLm1-2-6* (Fudal et al., 2007), and *AvrLm11* (Balesdent et al., 2013) have provided new understandings on how they are strategically located within regions that are rich in transposable elements, such that they can be lost or inactivated rapidly during sexual reproduction (Rouxel et al., 2011; Howlett et al., 2015). This fungal genomic structure is unique to *L. maculans* and often the effectors display little or no sequence similarity to each other (Van De Wouw et al., 2010; Rouxel et al., 2011). Specific recognition between *AvrLm4-7* and *Rlm4* and *Rlm7* genes and how the R and Avr effector proteins interact structurally has been examined using crystallography (Blondeau et al., 2015). There should be a focus on studies that provide additional molecular details at the gene level which would further our understanding of the factors leading to virulence and susceptibility associated with *R*-gene mediated resistance.

## Future work

Identifying *R* genes in *B. napus* against the major pathogens discussed here can be a formidable task, for example seeing the difference in physiological host resistance response between *Brassica* hosts in *P. brassicae*, strong genetic linkage between resistance and other agronomic traits in *S. sclerotiorum*, the recessive nature of *R* genes in *H. parasitica*, and nomenclature issue of *R* genes in *L. maculans*.

Efforts to identify *R* genes have been assisted by the development and application of differential host-pathogen pathotype/race/isolate combinations. Further improvements can be made by establishing freely-available universal host differential sets for each pathogen; differential sets that encompass the host resistances and pathogen sub-specific variation worldwide. One of the approaches that could be taken is an international collaborative network creating a world database for each pathogen. An example of this is the initiative established at the Brassica 2016 conference to resolve the Blackleg nomenclature issue (Plissonneau et al., 2017b). Since dynamic changes can happen with both host cultivars and pathogens in terms of cultivar resistance breakdowns and/or pathogens losing/gaining in terms of their pathogenicity/virulence over time, it is imperative that any universal database set up be regularly updated and kept current. For example, the international DivSeek, database, for phenotyptic data that can be linked with genotypic data (for seeds that are stored in seed bank), that was launched recently (Editorial, 2015) could be utilized as a model to set up the data bases needed.

Except for the four *R* genes that have been cloned, the QTL for the major diseases in *Brassica* have been identified but the causal genes remain unknown. Cloning of *R* genes has historically been undertaken using traditional methods, such as map-based cloning (Hatakeyama et al., 2013; Larkan et al., 2013). However, the recent genomics revolution has provided significant insights into the presence and absence of expressed genes in the *Brassica* A and C genomes using a pan-transcriptome approach (He et al., 2015). This, along with the recent pan-genome research (Golicz et al., 2016) readily allows cloning of *R* genes in crops such as *Brassica* that have complex genomes. The success story of the cloned *R* genes related to Clubroot and Blackleg tells us that the *R* genes in the *Brassica* host plant acting against different major pathogen can belong to different classes, not necessarily the common NBS-LRR class. More *R* gene cloning work in *Brassica* will help us better understand how the plant defends itself against these pathogens and we can use this knowledge to exploit novel *R* genes in a quicker and more effective manner for breeding purposes.

The impact of climate change on crop diseases has been increasingly addressed over recent decades (Fizgerald, 2016), making the characterization of new *R* genes more crucial than ever. MutRenSeq technology has been used to clone bread wheat stem rust resistance genes *Sr22* and *Sr45* (Steuernagel et al., 2016) and the potato late blight resistance gene, *Rpi* (Witek et al., 2016). This technique allows more rapid and precise cloning (Bent, 2016). For example, sequence-specific nucleases technology to target specific genes such as MILDEW-RESISTANCE LOCUS (MLO) in efforts to increase wheat (polyploid) resistance to Powdery Mildew have been undertaken (Gil-Humanes and Voytas, 2014; Wang et al., 2014). Such methodologies can now be similarly applied for precise alteration of disease resistance genes in *Brassica*.

Effective deployment of *R* genes in breeding *B. napus* resistant crops for enduring sustainability demands detailed knowledge about the ecology and life history of both host and pathogen and how their interactions may shape their evolution over time (Burdon and Thrall, 2009; Burdon et al., 2014). Such knowledge could for example, allow prediction of the host resistance at different stages of the infection processes for the pathogen, potentially allowing identification and targeting of the resistance gene(s) effective at the earliest infection stage in order to maximize impedance and/or even prevent the establishment of the pathogen and also curtail subsequent pathogen inoculum production and consequent secondary spread of the disease. While this approach could be particularly applicable for a disease such as Clubroot, there are still challenges as it is not yet possible to identify the co-relationship between root hair infection (primary) and root cortex infection (secondary) (Voorrips, 1995). On the other hand, knowledge about controlling pathogen infection at different developmental stages of the host (e.g., seedling vs. adult) or even in different plant organs (e.g., cotyledons, leaves, stems, pods, and root) where the pathogen infection and colonization has the greatest adverse impact on the productivity of the plant (e.g., plant stems for *L. maculans* and *S. sclerotiorum*) have become more forthcoming for the major pathosystems in *B. napus*. Clearly, improving our understanding of the mechanism(s) at both life-stages of the pathogen infection and colonization processes, the development stages of the host, and the host-pathogen interactions should open up new opportunities to better understand and exploit approaches that maximize control of pathogen infection and spread to minimize consequent yield losses.

Sequencing of *Brassica* genomes will allow new discoveries and understandings regarding the genetic relationships of different *R* genes, within and between members of the Brassicaceae. Although hybridization between divergent groups in the Brassicaceae, which contains rich resources of *R* genes, is already possible, particularly in India, this is an area of great future potential that is likely to lead to identification of many new *R* genes as these methodologies become more widely adopted and as rates of success with wide-hybridization events improve. While scientists are able to make informed decisions about utilizing race-specific and/or nonrace-specific resistance genes, further understanding of the benefits of different patterns of deployment for each type individually or rotation of both types, will identify better approaches toward maximizing the durability and effectiveness of both types of resistances, particularly *R* genes. Further, field deployment of race-specific and/or non-race-specific resistance genes will have a large impact on the dynamics within pathogen populations. Combining race-specific and race nonspecific genes together in the one cultivar will also potentially increase durability of *R* genes deployed within *Brassica* species, and has already been demonstrated for *R* genes against *L. maculans* (Brun et al., 2000, 2010; Delourme et al., 2014). Improved resistance from gene pyramiding through marker-assisted selection was reported for the *P. brassicae*-*B. rapa* ssp. *pekinensis* pathosystem (Matsumoto et al., 2012). However, this remains a controversial approach with some suggesting that such an approach could lead to an undesired outcome of breakdown of the multiple *R* genes pyramided (Essenberg et al., 2014). As we gain more knowledge about the molecular mechanisms of the plant immunity signaling pathway and the role of *R* genes, this will open up more effective and durable strategies to overcome pathogen attack (Swiderski and Innes, 2001; Cheng et al., 2015).

Although next-generation sequencing for pathogen effectors is progressing rapidly, and with large amounts of new knowledge becoming available for use from both host and pathogen viewpoints, the challenge now is to transfer this new knowledge from the laboratory into the field in order to enable better disease management. In this review, we have highlighted the challenges in identification and deployment of *R* genes, and evaluated options for the most effective utilization of *R* genes to improve resistance to the major fungal, oomycete or chytrid pathogens of *B. napus* with the support of genomic advances. These insights will help bring us closer toward breeding *B. napus* with improved and more durable disease resistances that secure the future of *B. napus* as a major food crop worldwide.

## Author contributions

TN conceptualized and drafted the manuscript, with additions and edits from JB and MB. The figures and tables were prepared by TN. All authors read and approved the final manuscript.

### Conflict of interest statement

The authors declare that the research was conducted in the absence of any commercial or financial relationships that could be construed as a potential conflict of interest.
